# Discovery of temperature-induced stability reversal in perovskites using high-throughput robotic learning

**DOI:** 10.1038/s41467-021-22472-x

**Published:** 2021-04-13

**Authors:** Yicheng Zhao, Jiyun Zhang, Zhengwei Xu, Shijing Sun, Stefan Langner, Noor Titan Putri Hartono, Thomas Heumueller, Yi Hou, Jack Elia, Ning Li, Gebhard J. Matt, Xiaoyan Du, Wei Meng, Andres Osvet, Kaicheng Zhang, Tobias Stubhan, Yexin Feng, Jens Hauch, Edward H. Sargent, Tonio Buonassisi, Christoph J. Brabec

**Affiliations:** 1 Helmholtz-Institute Erlangen-Nürnberg (HI-ERN), Erlangen, Germany; 2 Department of Materials Science and Engineering, Institute of Materials for Electronics and Energy Technology (i‐MEET), Friedrich‐Alexander‐Universität Erlangen‐Nürnberg, Erlangen, Germany; 3 Hunan Provincial Key Laboratory of Low-Dimensional Structural Physics and Devices, School of Physics and Electronics, Hunan University, Changsha, China; 4 Photovaltaic Research Laboratory, Massachusetts Institute of Technology, Cambridge, MA USA; 5 Department of Electrical and Computer Engineering, University of Toronto, Toronto, ON Canada

**Keywords:** Electronic devices, Solar cells

## Abstract

Stability of perovskite-based photovoltaics remains a topic requiring further attention. Cation engineering influences perovskite stability, with the present-day understanding of the impact of cations based on accelerated ageing tests at higher-than-operating temperatures (e.g. 140°C). By coupling high-throughput experimentation with machine learning, we discover a weak correlation between high/low-temperature stability with a stability-reversal behavior. At high ageing temperatures, increasing organic cation (e.g. methylammonium) or decreasing inorganic cation (e.g. cesium) in multi-cation perovskites has detrimental impact on photo/thermal-stability; but below 100°C, the impact is reversed. The underlying mechanism is revealed by calculating the kinetic activation energy in perovskite decomposition. We further identify that incorporating at least 10 mol.% MA and up to 5 mol.% Cs/Rb to maximize the device stability at device-operating temperature (<100°C). We close by demonstrating the methylammonium-containing perovskite solar cells showing negligible efficiency loss compared to its initial efficiency after 1800 hours of working under illumination at 30°C.

## Abstract (web summary)

Current view of the impact of A-site cation on the stability of perovskite materials and devices is derived from accelerated ageing tests at high temperature, which is beyond normal operation range. Here, the authors reveal the great impact of ageing condition on assessing the photothermal stability of mixed-cation perovskites using high-throughput robot system coupled with machine learning.

## Introduction

Perovskite materials have opened up new avenues for fabricating high-performance optoelectronic devices^1–9^, among which formamidinium-lead-triiodide (FAPbI_3_) based multi-cation perovskites are of intense interest due to their superior optoelectronic properties^1,5,7,8,10–24^. Current understanding of the impact of A-site cations – that incorporation of inorganic cations improve perovskite stability while organic cations (e.g. methylammonium (MA)) destabilize materials and devices – is mainly derived from accelerated ageing tests at high temperatures that are far beyond the standard device-operating temperatures (60–85 °C)^6–8,11–19^ (Table S1). In this work, we use high-throughput engineering combined with machine learning to analyze the stability of multi-cation perovskites. We find that the impact of the ratio of organic: inorganic cations is reversed when the aging temperature is reduced to below 100 °C. Specifically, organic cation (e.g. MA) is in fact, a stability-enhancer while inorganic cation (e.g. Cs/Rb) is a stability-killer below 100 °C. We define this phenomenon as stability reversal in perovskites, which is further translated to device stability.

## Results

### Workflow of the high-throughput robot system

**Fig. 1:**
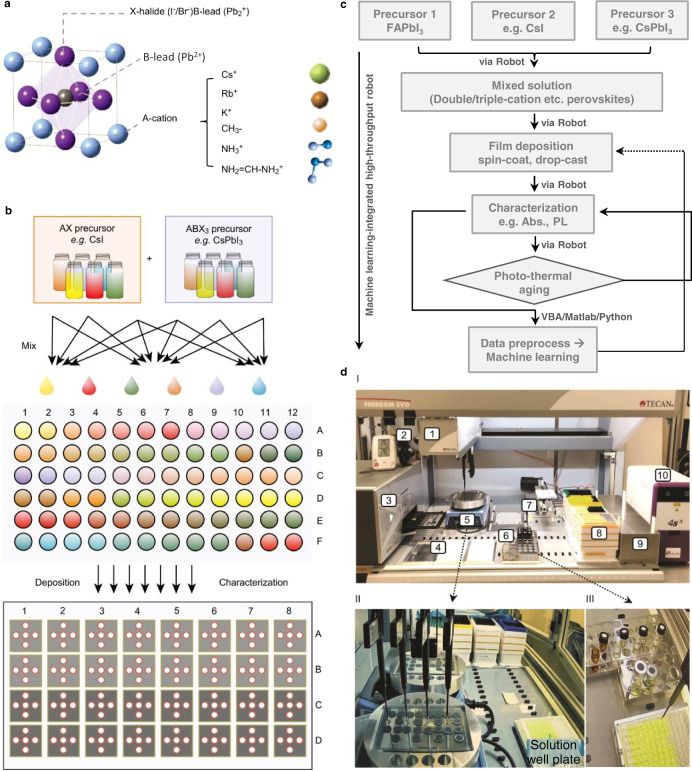
Workflow of automatic stability study via high-throughput robot (HTRobot). **a** Crystal structure of perovskite with multiple cations, including potassium (K^+^), rubidium (Rb^+^), caesium (Cs^+^), methylammonium (MA^+^), and formamidinium (FA^+^)**. b** Schematic of the workflow of HTRobot for automatic synthesis and characterization. The red circles in the bottom panel indicate the five different positions tested on each sample. **c** Detailed workflow chart of the high-throughput operation to evaluate perovskite stability. **d** Photograph of the HTRobot system, including (1) robot arm with four pipettes; (2) camera and humidity meter; (3) spectrometer to record the absorbance and photoluminescence; (4) 96-well microplates to mix the precursors; (5) hotplate; (6) stock solution of PbI_2_, FAI, MAI, CsI, etc.; (7) sample stage; (8) pipette tips; (9) waste container; and (10) heat sealer to optionally fuse microplates with aluminum foil. I/II/III show panoramic views of the setup, top view of the film fabrication and solution preparation, respectively.

First, we utilize a high-throughput robot (HTRobot) system coupled with machine learning to assess the photothermal stability of mixed-cation perovskites under different ageing conditions. The crystal structure of the ABX_3_ perovskite is shown in Fig. 1a, where A denotes a monovalent cation, B denotes lead, and X denotes a halide. In this study, FAPbI_3_ is the host material with methylammonium (MA), cesium (Cs), rubidium (Rb), and potassium (K) as combinatorial cations. As shown in Fig. 1b, the HTRobot system starts with a small number of mother solutions, followed by automatic mixing to form the desired precursors (e.g., FAPbI_3_). The as-prepared precursors were sequentially distributed to a custom 72-well plate and later used to deposit perovskite layer on glass substrates via drop-casting and spin-coating. For stability testing, the sample plates were transferred back-and-forth between the analytical platform and the climate chambers preset at varying temperature-humidity conditions (Supplementary Fig. 1). The detailed workflow is shown in Fig. 1c. The HTRobot automatically synthesizes, characterizes, and analyzes the materials of interest. The photographs in Fig. 1d, combined with Supplementary Movie 1, shows the platform component and its automatic process. In total, over 1000 samples were fabricated

### Stability analysis of mixed-cation perovskites

The figure-of-merit for stability assessment is denoted *T*_80_ = 0.2/$k_{{\mathrm{dec}}}$ and is obtained by the linear fitting of Eq. (1):

1 $$A = A_0 - k_{{\mathrm{dec}}} \times t$$

where *A* is the absorbance (normalized to 1), *t* is the aging time, and $k_{\mathrm{dec}}$ is the decomposition rate (s^−1^). *T*_80_ indicates the time required to decay 20% from its initial value^15^. During the decomposition process, the absorbance at 700–760 nm decreases. Instead of continuous decrease, the photoluminescence (PL) intensity shows an increasing trend at the early stage. This increase might be due to the improved crystal quality or PbI_2_ passivation^21,23^ (Supplementary Figs. 2 and 3). Since the relative absorbance value is only determined by perovskite degradation, we use the absorption spectra to extract *T*_80_ in the following discussion. This zero-order decomposition kinetics is also consistent with XRD measurement, showing a linear increase of PbI_2_ over time.

**Fig. 2:**
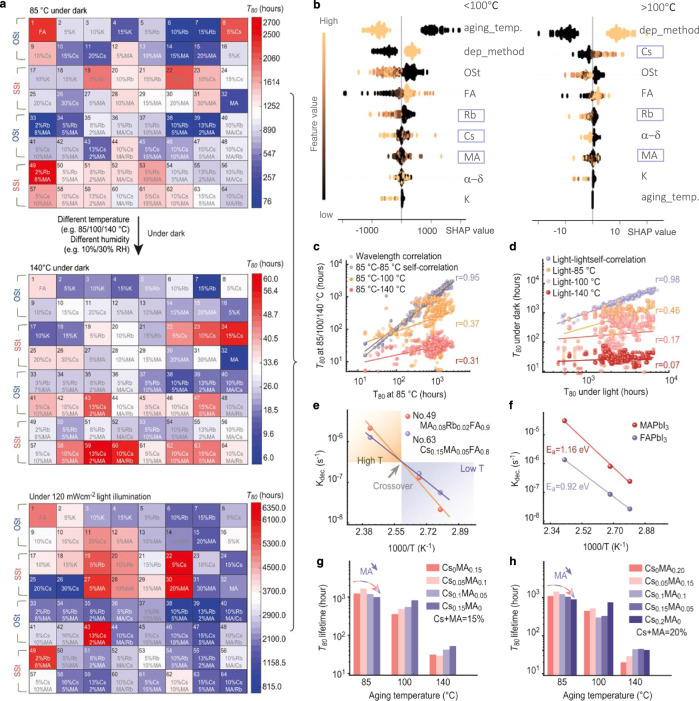
Lifetime analysis of the 64 compositions for mixed-cation lead iodide perovskites. **a** The colormap of T_80_ lifetime for the 64 perovskites aged in climate chambers preset at 85 °C (top) and 140 °C (middle) with 10% RH in the dark, and under 120 mW cm^−2^ metal-halide light illumination with N_2_ flow at 60 °C (bottom). **b** The ranking of feature importance using GBT regression and SHAP assessment, showing the impact of each cation and processing condition in descending order of importance (rank). The purple and orange color indicates low and high values of a given feature, respectively. Note that aging_temp., dep_method, Ost, α−δ indicates aging temperature, deposition method (drop-cast = 1), over-stoichiometric with excess iodide (OSt), the probability of phase transition in humid air (>30%RH), respectively. **c** The correlation plot of *T*_80_ at 85 °C against *T*_80_ at 85 °C, 100 °C, and 140 °C. Linear fitting was used to fit all the statistical data to obtain the Pearson correlation coefficients. **d** The correlation plot of *T*_80_ under light illumination against *T*_80_ aged at 85 °C, 100 °C, and 140 °C. **e** $k_{{\mathrm{dec}}}$-1000/T plot of No. 49 (MA_0.08_Rb_0.02_FA_0.9_PbI_3_) and No. 63 (Cs_0.15_MA_0.05_FA_0.9_PbI_3_). The data are fitted using Eq. (2), indicated by blue and red lines. **f** $k_{{\mathrm{dec}}}$-1000/T plot of MAPbI_3_ and FAPbI_3_. **g**, **h** *T*_80_ lifetime *versus* ageing temperature for a series of Cs_*x*_MA_*y*_FA_1*-x-y*_PbI_3_ perovskites, with *x* + *y* = 15% (**g**) and *x* + *y* = 20% (**h**).

Sixty-four kinds of perovskite including over stoichiometric samples with excess halide salts were fabricated through drop-cast and spin-coat methods^3^, as shown in Table S1. The selection for the 64 compositions is mainly based on the phase diagram of mixed-cation perovskites^25^. The selection for the 64 compositions is mainly based on the phase diagram of mixed-cation perovskites^25^. Above 300 K, the miscibility gap is at ~25 mol.% for Cs/MA cations in FAPbI3-based perovskites while it is at ~5 mol.% for K/Rb cations. To examine the de-mixing effect on photo/thermal stability of perovskites, we also prepared some perovskites with excess 5 or 10 mol.% MA/K/Rb/Cs above the miscibility gap. The XRD spectra show that most pristine samples mainly consist of α-phase perovskite (Supplementary Fig. 4), except for the control FAPbI_3_. Figure 2a presents the *T*_80_ color map for the 64 unsealed drop-cast films aged at 85 °C/140 °C with 10% RH and under illumination with N_2_ flow. Low humidity is used because many samples show δ phase under high humidity (over 30% RH; Supplementary Fig. 5). Considering all the ageing conditions for these 64 compositions, we fabricated over 1400 samples in this study (Supplementary Fig. 6), and the raw data and analysis codes are provided in the Supplementary Data 1–2 and Supplementary Software 1, respectively.

We first determined the features governing stability at different aging temperatures using gradient boosting decision tree (GBT) interpreted by SHapley Additive exPlanations (SHAP), as shown in Fig. 2b. The observation and prediction results of the randomly-split train-test set indicate high accuracy of GBT regression (Supplementary Fig. 7). A positive SHAP value indicates the beneficial impact of one feature on model output (predicted *T*_80_). The features ranked using a global database show that the stoichiometry and deposition method has large impacts on stability. The over-stoichiometric condition (Row. 1/2/5/6) leads to worse stability (Row. 3/4/7/8; Fig. 2a), which may be related to their higher defect density implied by the lower PL intensity (Supplementary Fig. 8). The influence of the deposition method is due to variations in the grain size (Supplementary Fig. 9), defect density, or internal strain^26,27^.

Remarkably, we discover that the impact of each feature on the stability is temperature-dependent, especially for the impact of cation. The incorporation of Cs shows beneficial impact on stabilizing perovskites at high temperatures (>100 °C) while it becomes detrimental at low temperatures. Differing from Cs, doping of MA is overall neutral to stabilizing perovskites and becomes more positive at low temperatures (<100 °C; Fig. 2b and Supplementary Fig. 7). The experimental reproducibility of our high-throughput system is validified by a close-to-one self-correlation coefficient by correlating *T*_80_ lifetime between two samples with identical compositions, processings and ageing conditions (Fig. 2c/d and Supplementary Fig. 10). By plotting *T*_80_ at 85 °C against *T*_80_ at 100 °C/140 °C for each identical composition, we further demonstrate a weak correlation between high/low-temperature stability manifested by low Pearson correlation coefficients of 0.37/0.31 (Fig. 2c). The above results indicate that low-temperature stability does not have a simple linear relationship with high-temperature stability. For photo-stability at 60 °C with N_2_ flow, the correlation between photo-stability and thermal-stability shows even lower Pearson coefficients of 0.07/0.17 when the aging temperature is over 100 °C (Fig. 2d). A low *r*-value of 0.35 is also observed in the spin-coating samples for 85 °C/140 °C correlation (Supplementary Fig. 12).

GBT regression of separated drop-cast/spin-coat subset further demonstrates the generic character of stability reversal in perovskites: higher ratio of MA to Cs, MA to Rb, or FA to Cs normally produces better stability below 100 °C while it produces worse stability above 100 °C (Supplementary Fig. 7). Meanwhile, by comparing the *T*_80_ of Cs/MA-containing samples, we find that 140 °C-*T*_80_ shows an increasing trend from No. 22 (Cs_0.05_FA_0.95_PbI_3_) to No. 26 (Cs_0.30_FA_0.70_PbI_3_), while this trend is almost reversed for 85 °C-*T*_80_ (Fig. 2a). No. 53 → 55, No. 57 → 59, No. 61 → 63, and No. 8 → 11 show similar reversal behaviors. The Arrhenius plot further shows that *k*_dec_ = 0.2/*T*_80_ of MA-rich perovskites grows faster than that of MA-poor perovskites (MA concentration <5%) as the ageing temperature rises, leading to a crossover at approximately 110 °C (Fig. 2e). This phenomenon is also observed in No. 28 vs. No. 22/23 with a crossover at ~85 °C (Supplementary Fig. 13). MAPbI_3_ (No. 32) also shows stability reversal with FAPbI_3_ (No. 1), indicated by the Arrhenius plot (Fig. 2f). An extrapolated crossover at ~15 °C is expected. The slope is correlated with the equivalent activation energy Eq. (2):

2 $$k_{{\mathrm{dec}}} = {{k}}_0 \times e^{ - \frac{{E_{\mathrm{a}}}}{{k_{\mathrm{B}}T}}}$$

where *k*_0_ is the rate constant (s^−1^), *E*_a_ is the activation energy (eV) representing the height of the reaction barrier, and *k*_B_*T* is the product of the Boltzmann constant and the temperature.

That is, the decomposition of MAPbI_3_ has a larger $E_a$ than that of FAPbI_3_ perovskite (1.16 eV vs. 0.92 eV). The instability of MAPbI_3_ mainly originates from its huge rate constant ($k_0$) compared with FAPbI_3_ (10^9.4^ s^−1^ vs. 10^5.3^ s^−1^). This result is consistent with a previous report that MAPbI_3_ has higher activation energy than that of FAPbI_3_ for decomposition^28^. Likewise, MA-rich perovskites have statistically higher $E_a$ (0.84–1.06 eV) and *k*_0_ (10^4.5^ s^−1^–10^7.1^ s^−1^) than do Cs/Rb-rich perovskites ($E_a$: 0.71–0.92 eV; $k_0$: 10^2.3^ s^−1^–10^5.6^ s^−1^), as shown in Supplementary Fig. 13.

The *T*_80_ of a series of Cs_*x*_MA_*y*_FA_1*-x-y*_PbI_3_ (*x* + *y* = const.) further illustrates the stability-reversal behavior (Fig. 2g, h). At 140 °C, *T*_80_ increases with more Cs into the perovskite lattice; yet this trend is altered or even reversed as temperature drops below 100 °C. The stability-reversal behavior was also confirmed by tracking PbI_2_ using X-ray diffraction (Supplementary Fig. 14). The spin-coated samples present the same behavior as drop-cast samples (Supplementary Fig. 12).

For the photo-stability at 60 °C, increasing Cs (>5 mol.%) impairs the stability while increasing MA improves the stability in multi-cation perovskites (Fig. 2g). The beneficial effects of MA over Cs/Rb on photo-stability were also found in spin-coated perovskites (Supplementary Fig. 15). Without any encapsulation, MA-containing perovskites show ultralong *T*_80_ values of 3500 hours and 6300 hours for spin-coated films and drop-cast films, respectively (Supplementary Fig. 16). Although increasing the Cs concentration in some cases gradually improves *T*_80_ in over-stoichiometric samples, this does not reflect an intrinsic property because the excess halide salts are found to serve as feedstock to reconstruct perovskite under light soaking, causing spectral shift (Supplementary Fig. 17). Meanwhile, most samples without excess organic salts show negilible PL shift during the degradation process, except for Cs-rich perovskites (Cs > 15 mol.%) fabricated throguh spin-coating process showing siginificant blueshift at 140 °C (Supplementary Fig. 18). This blueshift might be caused by Cs accumulation in the degraded films after MA/FA is volatilized.

### Theoretical explanation for the stability-reversal behavior

To elucidate the possible origin of the stability-reversal behavior, we performed first-principles calculations for multi-cation perovskites. Since the decomposition rate can be transformed from thermodynamic control to kinetic control as the ageing temperature falls^29–31^ (Supplementary Note 1), we theoretically studied this system from both of the thermodynamic (dissociation energy) and kinetic (activation energy) perspectives.

First, the energy costs of perovskite decomposition from APbI_3_ to AI and PbI_2_, with A = MA/FA/Cs, are presented in Supplementary Fig. 19, and the values reflect the thermodynamic stability at high temperatures. We find that MAPbI_3_ is least favorable thermodynamically. The incorporation of MA also leads to the lower thermodynamic stability of FAPbI_3_ compared with the beneficial effect of Cs doping. This result explains the detrimental impact of increasing the ratio of MA to Cs and of FA to Cs on the high-temperature stability.

**Fig. 3:**
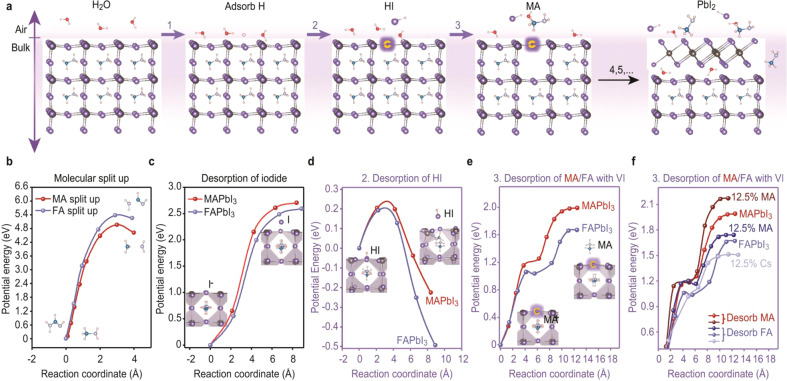
Decomposition path and the corresponding activation energy. **a** Schematic of the decomposition processes for perovskite with multiple steps, including proton adsorption (1st step), HI desorption (2nd step), MA/FA desorption (3rd step) and subsequent structure reconfiguration to form PbI_2_. **b** The kinetic energy barrier of MA/FA molecular splitting. **c**, **d** The potential energy curve *vs*. reaction coordinate for iodide desorption on the intact surface of MAPbI_3_/FAPbI_3_ and HI desorption on the surface of MAPbI_3_/FAPbI_3_ with an adsorbed proton. **e**, **f** The potential energy curve vs. reaction coordinate for MA/FA desorption on the surface of MAPbI_3_/FAPbI_3_ with iodide vacancies (**e**) and on the surfaces of Cs_0.125_ FA_0.875_PbI_3_ (12.5% Cs), MA_0.125_ FA_0.875_PbI_3_ (12.5% MA) with iodide vacancies (**f**). The orange circles indicate iodide vacancies. The two red curves represent MA desorption, and the three blue curves represent FA desorption.

To evaluate the stability at low temperatures, we examined the kinetic rate-limiting step by comparing the activation energies of several possible decomposition-pathways (Fig. 3a–d). Previous works suggested that MA splitting is the main reason for the poor stability of MA-containing perovskites^8,16,18,19^. However, the cleavage of MA has a large energy barrier of 5 eV, although it is smaller than that of FA cleavage (5.4 eV; Fig. 3b). We further examined the most accessible decomposition paths shown in Fig. 3a and the corresponding potential energy profiles (Fig. 3c–f). DFT results show that the protonation of surface I atoms plays a key role in promoting surface decomposition. With adsorbed protons, the activation energy for I-Pb bond-breaking and the subsequent HI desorption is reduced from ~2.7 eV to 0.2 eV for both MAPbI_3_ and FAPbI_3_. The protons can originate either from water molecules or from MA^+^/FA^+^ cations^32^. Subsequently, I vacancies at the Pb-I surface provide more feasible channels to desorb MA/FA molecules compared with the intact surface (Supplementary Fig. 19). The MA desorption barrier of 2.0 eV at the MAPbI_3_ surface is higher than that of FA (1.7 eV) at the FAPbI_3_ surface (Fig. 3e), which is consistent with the experimental results.

Theoretical simulations for doping systems also validate that the incorporation of MA/Cs could also affects the desorption energy of its surrounding FA organics at both nearest neighbor (Fig. 3f) and second nearest neighbor (Supplementary Fig. 20). Specifically, Cs decreases the desorption energy of its surrounding FA while MA shows the contrary impact. Combined with the thermodynamic results, we conclude that the incorporation of MA in FAPbI_3_ decreases its formation energy and simultaneously increases the gas desorption barrier, yet Cs exerts the opposite effects. The larger ${{k}}_0$ of MA-containing perovskites and MAPbI_3_ may also relate to the easier protonation of MA (Supplementary Note 2)^32^. To further validate our model, we identified the elements at perovskite surface using X-ray photoelectron spectroscopy with and without ion-beam etching (Supplementary Fig. 20). The results show that perovskites lose MA/FA cations after slight etching while Pb/I remains. Most interestingly, MA-containing perovskites tend to keep a higher organic cation concentration (MA/FA) at the film surface than FAPbI_3_ and Cs_0.15_FA_0.85_PbI_3_ under the same etching condition. Overall, DFT modeling and experimental results are in fairly good agreement and provide a plausible explanation of the temperature-induced stability reversal. However, we want to highlight that further DFT calculations are necessary and welcomed to further examine our model.

### Device stability of mixed-cation perovskites

**Fig. 4:**
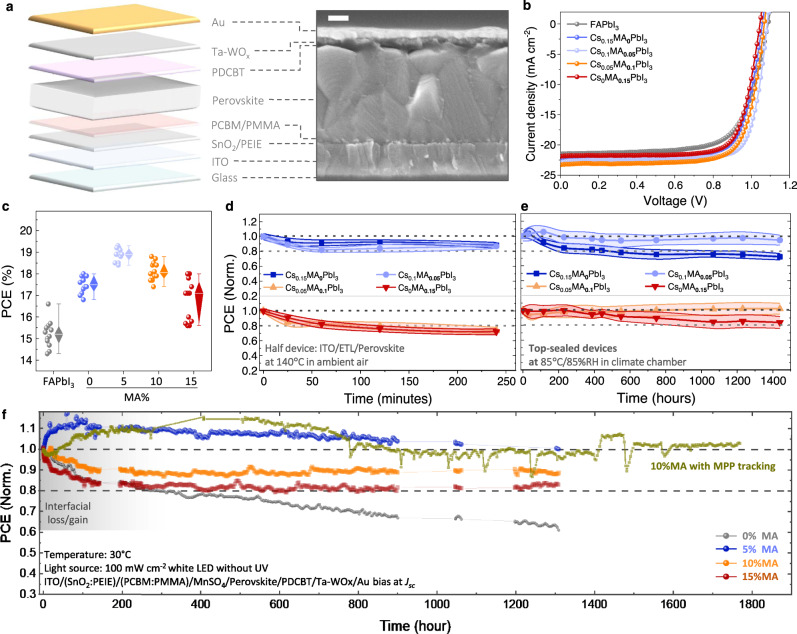
Device characterizations of Cs_*x*_MA_0.15–__*x*_FA_0.85_PbI_3_ perovskite solar cells. **a** Schematic of the solar cell architecture and the corresponding cross-sectional SEM image of the device. **b** Current density–voltage (*J*–*V*) curves for FAPbI_3_ and Cs_*x*_MA_0.15–__*x*_FA_0.85_PbI_3_ (*x* = 0, 5, 10, and 15%) perovskite solar cells with a scanning rate of 20 mV s^−1^ from −0.1 to 1.2 V. **c** Statistical PCE of 12 devices for each FAPbI_3_ and Cs_*x*_MA_0.15–__*x*_FA_0.85_PbI_3_ (*x* = 0, 5, 10, and 15%) perovskite solar cell. **d** Statistic PCE evolution under 140 °C/35%RH conditions in ambient air. The device was aged before depositing the hole transporting layer and Au. **e** Statistic PCE evolution under 85 °C/85%RH conditions in a climate chamber. **f** Long-term stability for Cs_*x*_MA_0.15–__*x*_FA_0.85_PbI_3_ (*x* = 0, 5, 10, and 15%) perovskite solar cells tested under 100 mW cm^−2^ white LED illumination. The efficiency was recorded with a scanning rate of 20 mV s^−1^ from 1.2 to −0.1 V as the reverse scan. The MPP tracking for 10% MA is based on the stabilized efficiency biased at the maximum power point in reverse-scan mode. The device temperature is controlled at ~30 °C by a cooling stage.

Combining phase stability with photo/thermal stability, the incorporation of appropriate MA with Cs/Rb/K (<5 mol.%) would maximize the device stability of FAPbI_3_-based perovskite solar cells (PSCs). We fabricated PSCs with mixed-cation perovskites (MA_*x*_Cs_0.15-*x*_FA_0.85_PbI_3_) with an n-i-p structure of ITO/(SnO_2_:PEIE)/(PCBM:PMMA)/MnSO_4_/perovskite/PDCBT/Ta-WOx/Au (Fig. 4a). The averaging power conversion efficiency (PCE) of MA-free perovskites was improved from 17.5% to 19.1% and 18.3% by incorporating 5% and 10% MA respectively, due to increased defect tolerance and carrier diffusivity^27,33^ (Fig. 4b, c). The performance parameters are summarized in Table S3. The hysteresis in current–voltage scan is negligible in this device structure (Supplementary Fig. 22).

The characterization of device stability follows the ISOS-D-2/3 protocol and ISOS-L-1 protocol^15^. Although MA-rich PSCs degrade faster than Cs-rich PSCs at 140 °C (Fig. 4d and Supplementary Fig. 23), the stability tests at standard device operating conditions did not follow this trend. Under 85 °C/85%RH, the top-sealed devices with Cs_0.15_FA_0.85_PbI_3_ suffer ~25% PCE loss after 1400 h of ageing, while the devices with 5–10% MA degrade less than 5% (Fig. 4e). None of the devices showed PCE losses after 2000 h of ageing at 60 °C in the dark (Supplementary Fig. 24). Note that the α−δ phase transition is inhibited by PDCBT layer and PEIE in SnO_2_.

We further tracked both the forward-scan and reverse-scan PCE of the devices biased at short-circuit conditions under 100 mW cm^−2^ white LED illumination, following the ISOS-L-1 protocol^15^. As shown in Fig. 4f, all the devices show complex degradation behavior, due to evolving interface and ions’ redistribution^34–36^. We found that the long-term degradation in the devices can be roughly divided into two regimes before and after ~250 h. In the initial ~250 h (regime I), the device degradation is non-linear, which might be attributed to the impact of contact layer and interfacial change. After ~250 h (regime II), it gradually follows a linear degradation law. In regime II, the degradation rate decreases with higher MA concentration for both the forward and reverse scans. The global evolution of absolute efficiency is presented in Supplementary Fig. 24. We further used maximum power point (MPP) tracking for one MA_0.1_Cs_0.05_FA_0.85_PbI_3_-based device. The device maintained 90% of the peak PCE value after 1800 h of continuous operation. The positive effect of MA on device operational stability at low temperatures is consistent with the high-throughput assessment of the photothermal stability of perovskite films. We also point out that the impact of perovskite composition on device stability can only be examined with robust contact layers in the device, otherwise the inactivation of contact or interface will dominate the overall device degradation (Supplementary Fig. 25).

## Discussions

Previous studies on the long-term stability of perovskites are mainly based on accelerated ageing experiments by comparing few samples’ thermal stability at high temperatures (Table S1). Cation engineering is an important strategy on this front: incorporation of inorganic cations into the perovskite lattice improves device stability, while organic cations (e.g. methylammonium (MA)) are viewed as destabilizing materials and devices. However, the study on device stability of perovskite solar cells with and without methylammonium shows contradictory results (Table S4). Based on automated high-throughput experiments under multiple ageing conditions, the importance of the specific temperature regime – in which the device will operate – is demonstrated. The effect of one cation on stability can be switched between detrimental and beneficial when the ageing condition is switched between low temperature and high temperature. The mechanism underlying the stability reversal is the change of both activation energy and rate constant in the decomposition by incorporating multiple cations into the perovskite lattice. This work sheds light on the complexity behind perovskite decomposition from the kinetic (activation energy) perspective, although we only provide one possible mechansim to explain the stability reversal. The stability reversal is only an icerberg of complex phenomena behind perovskite decomposition, for which further experimental and theoretical investigation on perovskite instability are still required. We note that the decomposition rate is non-linear with ageing temperature in the kinetic equation. Moreover, the activation energy and rate constant can be changed by manipulating the composition. With ageing temperature increases over a critical point, the relationship of stability between different perovskites could be very different, which is manifested by the low Pearson correlation coefficient. That is, a simple mapping relation between high- and low-temperature stability for perovskite films is unreliable, at least for all-iodine perovskites. A broader high-throughput study on cation engineering in I/Br-mixed perovskites is expected to reveal more helpful strategies to improve perovskite stability in the future. Based on our results, new strategies – incorporation of at least 10 mol.% organic MA and up to 5 mol.% inorganic cations (Cs/Rb) in perovskite lattice – should be adopted to improve the stability of device applications at temperatures below 100 °C. Considering the *T*_80_ lifetime for some screened perovskite is far beyond 4000 h under 120 mW cm^−2^ illumination at 60 °C, the stability of contact layers in devices is lagging behind the film stability for most reported devices. In addition to seeking ultra-stable perovskite layers, we should pay more attention to develop stable contacts to achieve longevity of perovskite photovoltaics.

## Methods

### Materials and solution preparation

Unless stated otherwise, all materials were purchased from Sigma Aldrich or Merck and used as received. MAI and FAI were purchased from Xi’an P-OLED. PbI_2_ was purchased from Lumtec. The Ta-WOx colloidal solution was purchased from Avantama Ltd. The SnO_2_-PEIE solution was prepared by mixing 15 wt% SnO_2_ aqueous solution (300 µL) with 1.8 mL of isopropanol and H_2_O (1/1, v/v) and 20 μL of PEIE. The PMMA:PCBM (1:5) precursor solution was prepared by mixing 1 mg/mL PMMA and 5 mg/mL PCBM chlorobenzene solution (1/1, v/v). For high-throughput film fabrication, a 1-M PbI_2_/FAI solution was prepared first in DMF and DMSO (4:1 v/v). A 1-M MAI/CsI/RbI/KI solution was prepared in DMSO. Sixty-four mixed-cation perovskite precursors were obtained by mixing the above mother solutions at the specific ratios described in Supplementary Table 1. Here, the molar ratio is based on the volume ratio of the mother solutions. For film fabrication in devices, 1.2 M PbI_2_/FAI solution was prepared first in DMF and DMSO (4:1 v/v), and 1.2 M MAI/CsI solution was prepared in DMSO. The MA_*x*_Cs_*y*_FA_(1-*x-y*)_PbI_3_ precursors were prepared by mixing the mother solutions in the target ratio. The PDCBT was dissolved in chlorobenzene at 15 mg/mL and stirred at 80 °C for 10 min before use. The PCBM was dissolved in chlorobenzene and 1,2-dichlorobenzene (9:1 v/v) at 10 mg/mL and stirred at 80 °C for 10 min before use. The MnAc_2_ and (NH_4_)_2_SO_4_ solutions (1.5 mg/mL) were prepared in a mixed solvent (H_2_O/IPA = 1/1, v/v).

### Film fabrication

The high-resolution video for the high-throughput fabrication using drop-casting and spin-coating can be found in https://data.mendeley.com/datasets/6rbpx2fxf7/draft?a=a8ecd292-6a04-4467-9e38-85f30d937f5c and https://data.mendeley.com/datasets/9y74p4pyjz/draft?a=ce49c6e3-e3be-4e2b-9dda-41a4cdb5249e. For drop-casting, the precursors were diluted to 0.3 M using DMF to control the film thickness to a few hundred nanometers. The hotplate was kept at 130 °C, and a 4-μL aliquot was dropped on the plasma-treated glass substrate on the hotplate via the high-throughput robot system. After the program was finished, the samples were transferred to a N_2_-filled glovebox for post-treatment at 150 °C for 5 min before measurement. For each batch of sample, it takes around 3 min to finish the depostion process. For spin-coating, the plasma-treated glass substrates were coated with 80 μL of perovskite solution (1.2 M) at 200 rpm for 2 s, 2000 rpm for 2 s and 5000 rpm for 40 s. During the last step, 180 μL of chlorobenzene was dropped on the film at 20 seconds, followed by annealing at 110 °C for 10 min and 150 °C for 5 min. Since our home-made spin-coating robot has not been enclosed by N_2_-filled glovebox, the processing in ambient air with prolonged time might pose an issue for the perovskite precursors and anti-solvent. Therefore, the spin-coat samples for high-throughput characterization are based on manual fabrication in N_2_-filled glovebox using the robot-prepared precursors.

### Analysis of the high-throughput data

The data classification was processed by home-made VBA codes, while the data fitting is realized by VBA codes and MATLAB codes. The related information can be found in the auxiliary materials and Supplementary Movie 1.

The raw dataset is uploaded to

10.17632/j3c7wnwxbc.1

The analysis codes are uploaded to

10.17632/2rbp5x8hf7.1

10.17632/42zcttrbr9.1

### Device characterization

The *J*–*V* curves of the solar cells were obtained using a Keithley source under 100 mW cm^−2^ AM1.5 G illumination (Newport SollA solar simulator). The light intensity was calibrated with a certified crystalline Si-cell. The area of aperture mask is 0.113 cm^2^. The *J*–*V* characteristics were measured from −0.1 to 1.2 V (forward scan) and 1.2 to −0.1 V (reverse scan) at a scan rate of 20 mV/s. The tests were tested in air at room temperature (around 25 °C). The external quantum efficiency (EQE) spectra were taken using an Enli Technology system. For the stability test, illumination was provided by a white LED (XLamp CXA2011 1300 K CCT) with 100 mW cm^−2^ intensity. The testing chamber was sealed by glass cover and filled with fresh N_2_ gas. The temperature is controlled at ~27 °C during the testing period. For maximum power point (MPP) tracking, the MPP tracking point is based on the reverse *J*–*V* scan from 1.2 to −0.1 V every 2–3 h. The stabilized efficiency was obtained by fixing the bias at the MPP point for 2 min. The device temperature is controlled at ~30 °C by a cooling stage under the chamber. For the thermal stability test at 60 °C, the devices were placed on a hotplate kept at 60 °C without any encapsulation in ambient air. The humidity ranged from 35% to 45% RH. For the thermal stability test at 85 °C, the devices were top-sealed with glass cover and stored in a sample box in a climate chamber in the dark. For the thermal stability test at 140 °C, half devices without a hole transporting layer were placed on a hotplate in ambient air without any encapsulation. After the ageing process, the half devices were transferred to a glovebox for subsequent deposition of the hole transporting layer and Au.

### SHAP analysis based on Gradient-Boosted Trees

The SHAP (SHapley Additive explanation) analysis follows the protocol from our previous work^37^. First, the dataset is grouped into three categories (<100 °C, 100 °C, and >100 °C) and is further split into 80%: 20% train: test set before being trained on gradient boosting with decision trees regression on scikit-learn python package^38^. The hyperparameters are optimized in the aforementioned algorithm to have the minimum 5-fold cross-validated root mean square error (RMSE). There are nine features included in the dataset: the compositions of *A*-site cations (K, Rb, Cs, MA, and FA), the deposition method (spin-coating or drop-casting), the stoichiometry (high value for over stoichiometry, and low value for standard stoichiometry), alpha-delta transition (high value indicates the presence of δ-phase in the pre-degraded films, and vice versa), and aging temperature (60, 85, 100, or 140 °C).To interpret the results and see how each dataset’s features contribute to the *T*_80_ output, the Shapley values from the trained models are analyzed using SHAP (SHapley Additive explanation) package^37^.

Each point in the SHAP plot represents the measured data point. High SHAP value means that the data point has a high *T*_80_ value and vice versa. The SHAP plot is ranked by its importance, with the top one represents the most important feature that contributes to the output. The color bar represents how high and low the feature values are. Based on this information, we can see how each feature value contributes to a higher or lower *T*_80_ value based on the trained model^39^.

### Computational methods

DFT calculations: The theoretical simulations were performed within the density functional theory (DFT) method as implemented in the Vienna ab initio simulation package (VASP) code. We used the Perdew, Burke and Ernzerhof generalized gradient approximation (PBE-GGA) for the exchange correlation functional. The atomic structures were fully relaxed until the maximum force acting on each ion was <0.01 eV Å^−1^. The 2 × 2 × 2 supercells were adopted with Monkhorst–Pack (MP) k-point meshes of 3 × 3 × 3 for bulk calculations. For all the considered perovskites, we used the cubic phases (a = b = c) in our simulations. The lattice constants are listed in Supplementary Table 3. For the perovskite surface, we employed the model with the bottom layer atoms fixed to their bulk positions and a vacuum space of 15 Å was imposed to avoid interactions between adjacent units. The energy profiles for decomposition of the 001 surface of MAPbI_3_ and FAPbI_3_ were calculated with the climbing image nudged elastic band (CI-NEB) method.

### Reporting summary

Further information on research design is available in the Nature Research Reporting Summary linked to this article.

## Supplementary information

Supplementary Information

Description of Additional Supplementary Files

Supplementary Data 1

Supplementary Data 2

Supplementary Movie 1

Supplementary Software 1

Solar Cells Reporting Summary

## Data Availability

The high-throughput data for the thermal-/photo-stability are available in separate Supplementary Data files in the Supplementary Information section. All other relevant data are available from the corresponding authors upon reasonable request.

## Code availability

All the codes used in this manuscript can be found in:

10.17632/2rbp5x8hf7.1

10.17632/42zcttrbr9.1

https://github.com/PV-Lab/capping-layer
