# Supplementary material for: Discovery of temperature-induced stability reversal in perovskites using high-throughput robotic learning
Source: Nat Commun. 2021 Apr 13;12:2191. doi: 10.1038/s41467-021-22472-x (PMC8044090; doi:10.1038/s41467-021-22472-x)
Supplement: Supplementary file 1 — Supplementary Information [file 41467_2021_22472_MOESM1_ESM.pdf]

Supplementary information

Discovery of temperature-induced stability reversal in perovskites using high-throughput robotic learning

Y. Zhao et al.

Corresponding to:
yicheng.zhao@fau.de
yexinfeng@pku.edu.cn;
christoph.brabec@fau.de

Supplementary methods

Device fabrication

First, ITO substrates were sonicated in acetone and isopropanol for 30 minutes and 60 minutes, respectively. SnO_2-PEIE solution (80 μL) was spin-coated on ITO substrates at 3500 rpm for 30 seconds and then annealed at 150°C for 10 minutes in ambient air. Then, 80 μL of PCBM:PMMA solution was spin-coated on a SnO_2/PEIE layer at 2000 rpm for 30 seconds and then annealed at 150°C for 10 minutes in a glovebox. $MnSO_4$ modification was achieved by sequentially spin-coating $MnAc_2$ and $(NH_4)_2SO_4$ solutions at 2000 rpm/30 s and annealing each layer at 150°C for 10 minutes in a N_2-filled glovebox. Perovskite solution (1.2 M, 80 μL) was spin-coated on the $MnSO_4$-modified substrate by using the following parameters: 200 rpm for 2 seconds, 2000 rpm for 2 seconds, and 5000 rpm for 40 seconds (a=3 seconds). Then, 180 μL of chlorobenzene was dropped on the film at 20 seconds, followed by annealing at 110°C for 10 minutes and 150°C for 5 minutes. The as-prepared perovskite film was then spin-coated by PDCBT as a hole transporting layer at 2000 rpm for 40 seconds and annealed at 90°C for 5 minutes. Finally, 100 μL of Ta-WOx was coated on PDCBT at 2000 rpm for 30 seconds and annealed at 75°C in ambient air. A 100-nm-thick Au electrode was deposited through a shadow mask *via* thermal evaporation. For the devices used in the stability tests, a 200 nm Au layer was deposited.

Film characterization

High-throughput PL/Abs. characterization was performed with TECAN infinite 200Pro. The PL signal was collected from the top side of the perovskite film from 650 nm to 850 nm with a 4 nm step. The absorbance signal was collected from 720 nm to 850 nm with a 2 nm step. Scanning electron microscopy (SEM) images were obtained by using a 10 kV acceleration voltage with an FEI Helios Nanolab 660 setup. X-ray diffraction analysis was performed with Bragg-Brentano geometry using a Panalytical X'pert powder diffractometer with filtered Cu-Kα radiation and an X'Celerator solid-state stripe detector.

Supplementary Note 1

A simplified model for thermodynamic control vs. kinetic control

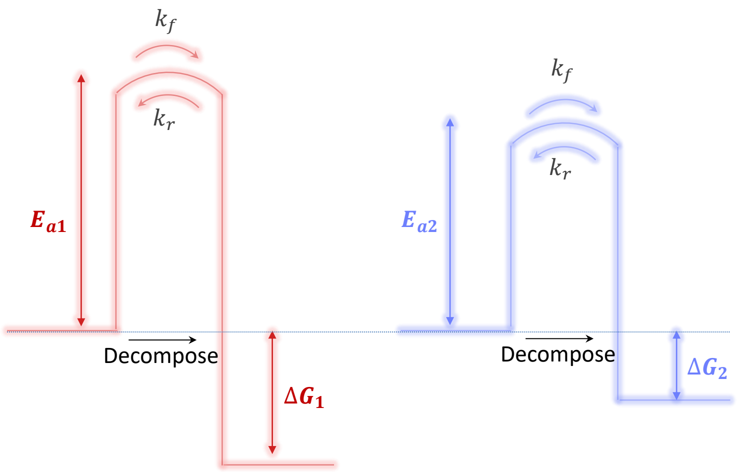

Fig. N1 Schematic of the potential diagram for the decomposition of two materials: one has both a higher activation energy (E_a) and dissociation energy (ΔG) than the other.

The potential decomposition rate is mainly influenced by two factors: 1. the relative stability of the decomposition product, which is associated with ΔG; and 2. the energy barrier of the decomposition, which is associated with E_a. The following equations provide a basis for understanding the transformation from thermodynamic to kinetic control.

Decomposition rate Δk_1 of reaction 1:

$$\Delta k_1 = k_{f1} - k_{r1}$$

$$= exp\left(-\frac{E_{a1}}{k_B T}\right) \times (k_{f0} - k_{r0} \times exp\left(-\frac{\Delta G_1}{k_B T}\right))$$

$$= k_{01}^{eff} exp\left(-\frac{E_{a1}}{k_B T}\right) \qquad (1)$$

Decomposition rate Δk_2 of reaction 2:

$$\Delta k_2 = k_{f1} - k_{r1}$$

$$= exp\left(-\frac{E_{a2}}{k_B T}\right) \times (k_{f0} - k_{r0} \times exp\left(-\frac{\Delta G_2}{k_B T}\right))$$

$$= k_{02}^{eff} exp\left(-\frac{E_{a2}}{k_B T}\right) \qquad (2)$$

The relative rate r is derived as:

$$r = \frac{\Delta k_1}{\Delta k_2}$$

$$= \frac{k_{01}^{eff} exp\left(-\frac{E_{a1}}{k_B T}\right)}{k_{02}^{eff} exp\left(-\frac{E_{a2}}{k_B T}\right)}$$

$$= \exp\left(-\frac{\Delta E}{k_B T}\right) \times \left(\frac{k_{01}^{eff}}{k_{02}^{eff}}\right) \qquad (3)$$

where $k_{f1/2}$ and $k_{r1/2}$ indicate the forward and backward reaction rates, respectively. k_{01}^{eff} and k_{02}^{eff} denote the effective pre-exponential factor in the Arrhenius equations (1) and (2), respectively. $\Delta E = E_{a1} - E_{a2}$. Reaction 1 will have a lower decomposition rate than reaction 2 if $r < 1$.

Considering the volatile nature of MAI/FAI, the backward reaction constant k_{r0} is much lower than k_{f0}, and the right hand sides of equations (1), (2), and (3) are positive in the decomposition process.

At temperature falls, k_{01}^{eff} and k_{02}^{eff} become constant, and $\exp\left(-\frac{\Delta E}{k_B T}\right)$ dominates the decomposition rate in equation (3). In this case, the decomposition is kinetically controlled, which is dictated by the relative activation energy. Since $\Delta E > 0$, the first material will have a larger decomposition rate than the second one below a critical temperature.

Supplementary Note 2

Possible origins of the different pre-exponential factors

The decomposition process from perovskite to PbI_2 is a multistep process. The protonation of surface iodide is the first step, leading to an iodide vacancy. We speculate that the frequency of protonation plays a key role in determining the reaction constant K_o in the Arrhenius equation, in addition to ΔG. The protons can originate from water molecules in the environment or MA^+/FA^+ in the perovskite lattice.

With H_2O, the hydrolysis rate at the $MAPbI_3$ surface is higher than that at the $FAPbI_3$ surface, leading to a larger K_0 for $MAPbI_3$ or MA-containing perovskites:

$$H_2O + CH_3NH_3PbI_3 \rightarrow H^+ + OH^- + CH_3NH_3PbI_3 \rightarrow CH_3NH_3PbI_3 \cdots H^+ + OH^-$$

$$\rightarrow CH_3NH_3PbI_2^+ + HI + OH^- \rightarrow PbI_2 + HI + CH_3NH_3^+ \cdots OH^-$$

Without H_2O, the higher deprotonation/migration rate of MA^+ than FA^+ in perovskite leads to a larger K_0 for $MAPbI_3$ or MA-containing perovskites:

$$CH_3NH_3PbI_3 \rightarrow CH_3NH_2PbI_3^- \cdots H^+ \rightarrow CH_3NH_2PbI_2 + I^- \cdots H^+ \rightarrow PbI_2 + CH_3NH_2 + HI$$

Extended figure/table legends

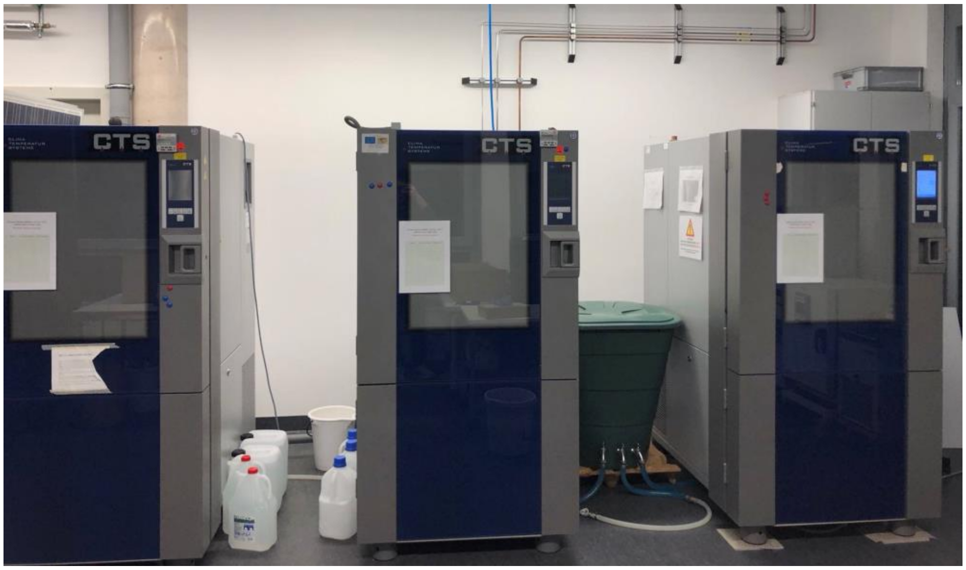

Supplementary Fig. 1. Three climate chambers for the thermal stability test under dark conditions.

Supplementary Table 1. A summary of previous studies on the film stability of mixed-cation perovskites.

Supplementary Table 1. Summary of studies on film stability of perovskites (MA^+: $CH_3NH_3^+$; FA^+: $NH_2CHNH_2^+$)

Aging condition	Composition and processing	Conclusion	Reference
130°C-dark in air (humidity not indicated)	$MAPbI_3$, $Cs_{0.05}(MAFA)_{0.95}PbI_3$, $MAFAPbI_3$, $Rb_{0.05}Cs_{0.05}(MAFA)_{0.9}PbI_3$, $FAPbI_3$, $Rb_{0.05}FA_{0.95}PbI_3$, $Cs_{0.1}FA_{0.9}PbI_3$, $Rb_5Cs_{10}FAPbI_3$ films *via* 1-step spin-coating	Organic MA deteriorates stability	Science 362, 449–453 (2018)
130°C-dark in air (humidity not indicated)	$Cs_x(MA_{0.17}FA_{0.83})_{(100-x)}Pb(I_{0.83}Br_{0.17})_3$ (x=0/10) films *via* 1-step spin-coating	Inorganic Cs improves stability	Energy Environ. Sci. 9, 1989-199 (2016)
140°C-dark in air (humidity: 40%RH)	$Cs_x(MA_{0.17}FA_{0.83})_{(100-x)}Pb(I_{0.97}Br_{0.03})_3$ (x=0/1/2/3) films *via* 2-step spin-coating	Inorganic Cs improves stability	Nat. Commun. 9, 1607 (2018)
130°C-dark in N_2	$FA_{0.83}Cs_{0.17}Pb(I_{0.6}Br_{0.4})_3$, $MAPb(I_{0.6}Br_{0.4})_3$ films *via* 1-step spin-coating	Higher stability of CsFA-based perovskites than MA	Science 351,151–155 (2016)
150°C-dark in N_2	$MAPbI_3$, $FAPbI_3$ films *via* 1-step spin-coating	MA deteriorates stability	Energy Environ. Sci. 7, 982-988 (2014)
150°C-dark in air	$MAPbI_3$, $FAPbI_3$ films *via* 1-step spin-coating	MA deteriorates stability	Nat. Nanotechnol 10, 391-402 (2015)

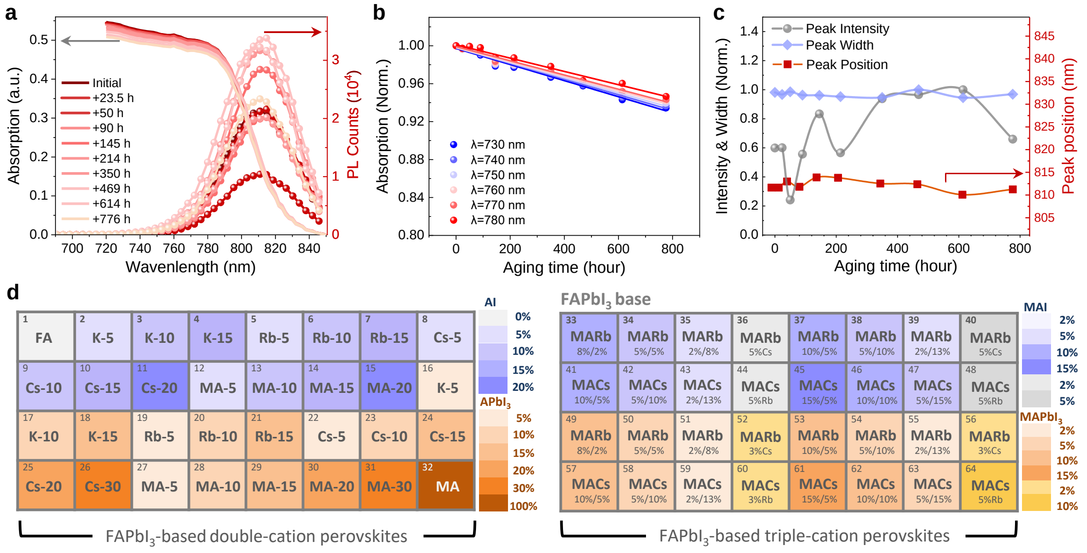

Supplementary Fig. 2. a The absorption/PL spectra *versus* ageing time on the timescale of hours for $FAPbI_3$ films fabricated through the drop-cast method under 85 ℃ and 10% RH. **b** The extracted absorbance values at six different wavelengths (from 730 nm to 780 nm) as a function of ageing time. **c** The PL intensity, peak position and full width at half maximum as a function of ageing time. These values were obtained by Gaussian fitting. The large perturbation of PL intensity over time is mainly caused by the small change inside the PL system during the long-term operation period. **d** Compositions of the mixed-cation perovskites studied in this work. The serial number at the top-left corner represents the different cation combinations, while the background colour indicates the doping concentration in the $FAPbI_3$ matrix. The left table mainly summarizes double-cation perovskites, and the right table summarizes triple-cation perovskites. For each combinatorial cation, both standard-stoichiometric and over-stoichiometric samples were prepared, and these are marked by orange and blue in the table, respectively.

Supplementary Table 2. A summary of the compositions of the 64 materials used in this study and their preparations.

Supplementary Table 2. Summary of the 64 composites and their corresponding recipes.

Serial Number	Target Composition	Recipe	Serial Number	Target Composition	Recipe
No.1	$FAPbI_3$	200 μL FAPbI3	No.33	$FAPbI_3$ + 2%RbI + 8%MAI	5 μL RbI +15 μL MAI + 200 μL FAPbI3
No.2	$FAPbI_3$+5%KI	10 μL KI + 200 μL FAPbI3	No.34	$FAPbI_3$+5%RbI+5%MAI	10 μL RbI +10 μL MAI + 200 μL FAPbI3
No.3	$FAPbI_3$+10%KI	20 μL KI + 200 μL FAPbI3	No.35	$FAPbI_3$+8%RbI+2%MAI	16 μL RbI + 4 μL MAI + 200 μL FAPbI3
No.4	$FAPbI_3$+15%KI	30 μL KI + 200 μL FAPbI3	No.36	$FAPbI_3$+5%RbI+3%CsI+2%MAI	10 μL RbI + 6 μL CsI + 4 μL MAI + 200 μL FAPbI3
No.5	$FAPbI_3$+5%RbI	10 μL RbI + 200 μL FAPbI3	No.37	$FAPbI_3$+5%RbI+10%MAI	10 μL RbI + 20 μL MAI + 200 μL FAPbI3
No.6	$FAPbI_3$+10%RbI	20 μL RbI + 200 μL FAPbI3	No.38	$FAPbI_3$+10%RbI+5%MAI	20 μL RbI + 10 μL MAI + 200 μL FAPbI3
No.7	$FAPbI_3$+15%RbI	30 μL RbI + 200 μL FAPbI3	No.39	FAPbI3+12.5%RbI+2.5%MAI	25 μL RbI + 5 μL MAI + 200 μL FAPbI3
No.8	$FAPbI_3$+5%CsI	10 μL CsI + 200 μL FAPbI3	No.40	$FAPbI_3$+5%RbI+5%CsI+5%MAI	10 μL RbI + 10 μL CsI + 10 μL MAI + 200 μL FAPbI3
No.9	$FAPbI_3$+10%CsI	20 μL CsI + 200 μL FAPbI3	No.41	$FAPbI_3$+5%CsI+10%MAI	10 μL CsI + 20 μL MAI + 200 μL FAPbI3
No.10	$FAPbI_3$+15%CsI	30 μL CsI + 200 μL FAPbI3	No.42	$FAPbI_3$+10%CsI+5%MAI	20 μL CsI + 10 μL MAI + 200 μL FAPbI3
No.11	$FAPbI_3$+20%CsI	40 μL CsI + 200 μL FAPbI3	No.43	$FAPbI_3$+13%CsI+2%MAI	26 μL CsI + 4 μL MAI + 200 μL FAPbI3
No.12	$FAPbI_3$+5%MAI	10 μL MAI + 200 μL FAPbI3	No.44	$FAPbI_3$+3%RbI+10%CsI+2%MAI	6 μL RbI + 20 μL CsI + 4 μL MAI + 200 μL FAPbI3
No.13	$FAPbI_3$+10%MAI	20 μL MAI + 200 μL FAPbI3	No.45	$FAPbI_3$+5%CsI+15%MAI	10 μL CsI + 30 μL MAI + 200 μL FAPbI3
No.14	$FAPbI_3$+15%MAI	30 μL MAI + 200 μL FAPbI3	No.46	$FAPbI_3$+10%CsI+10%MAI	20 μL CsI + 20 μL MAI + 200 μL FAPbI3
No.15	$FAPbI_3$+20%MAI	40 μL MAI + 200 μL FAPbI3	No.47	$FAPbI_3$+15%RbI+5%MAI	30 μL CsI + 10 μL MAI + 200 μL FAPbI3

No.16	$(K_{0.05}FA_{0.95})PbI_3$	10 µL KPbI3 + 190 µL FAPbI3	No.48	$FAPbI_3$+5%RbI+10%CsI+5%MAI	10 µL RbI + 20 µL CsI+10 µL MAI+ 200 µL FAPbI3
No.17	$(K_{0.1}FA_{0.9})PbI_3$	20 µL KPbI3 + 180 µL FAPbI3	No.49	$(Rb_{0.02}MA_{0.08}FA_{0.9})PbI_3$	4 µL MAPbI3 +16 µL MAPbI3 + 180 µL FAPbI3
No.18	$(K_{0.15}FA_{0.85})PbI_3$	30 µL KPbI3 + 170 µL FAPbI3	No.50	$(Rb_{0.05}MA_{0.05}FA_{0.9})PbI_3$	10 µL RbPbI3 + 10 µL MAPbI3 + 180 µL FAPbI3
No.19	$(Rb_{0.05}FA_{0.95})PbI_3$	10 µL RbPbI3 + 190 µL FAPbI3	No.51	$(Rb_{0.08}MA_{0.02}FA_{0.9})PbI_3$	16 µL RbPbI3 + 4 µL MAPbI3 + 180 µL FAPbI3
No.20	$(Rb_{0.1}FA_{0.9})$PbI3	20 mL RbPbI3 + 180 µL FAPbI3	No.52	$(Rb_{0.05}Cs_{0.03}MA_{0.02}FA_{0.9})PbI_3$	10 µL RbPbI3 + 6 µL CsPbI3 + 4 µL MAPbI3 + 180 µL FAPbI3
No.21	$(Rb_{0.15}FA_{0.85})PbI_3$	30 µL RbPbI3 + 170 µL FAPbI3	No.53	$(Rb_{0.05}MA_{0.1}FA_{0.85})PbI_3$	10 µL RbPbI3 + 20 µL MAPbI3 + 170 µL FAPbI3
No.22	$(Cs_{0.05}FA_{0.95})PbI_3$	10 µL CsPbI3 + 190 µL FAPbI3	No.54	$(Rb_{0.1}MA_{0.05}FA_{0.85})PbI_3$	20 µL RbPbI3 + 10 µL MAPbI3 + 170 µL FAPbI3
No.23	$(Cs_{0.1}FA_{0.9})PbI_3$	20 µL CsPbI3 + 180 µL FAPbI3	No.55	$(Rb_{0.13}MA_{0.02}FA_{0.85})PbI_3$	25 µL RbPbI3 + 5 µL MAPbI3 + 170 µL FAPbI3
No.24	$(Cs_{0.15}FA_{0.85})PbI_3$	30 µL CsPbI3 + 170 µL FAPbI3	No.56	$(Rb_{0.1}Cs_{0.03}MA_{0.02}FA_{0.85})PbI_3$	10 µL RbPbI3 + 10 µL CsPbI3 + 10 µL MAPbI3 + 170 µL FAPbI3
No.25	$(Cs_{0.2}FA_{0.8})PbI_3$	40 µL CsPbI3 + 160 µL FAPbI3	No.57	$(Cs_{0.05}MA_{0.1}FA_{0.85})PbI_3$	10 µL CsPbI3 + 20 µL MAPbI3 + 170 µL FAPbI3
No.26	$(Cs_{0.3}FA_{0.7})PbI_3$	60 µL CsPbI3 + 140 µL FAPbI3	No.58	$(Cs_{0.1}MA_{0.05}FA_{0.85})PbI_3$	20 µL CsPbI3 + 10 µL MAPbI3 + 170 µL FAPbI3
No.27	$(MA_{0.05}FA_{0.95})PbI_3$	10 µL MAPbI3 + 190 µL FAPbI3	No.59	$(Cs_{0.13}MA_{0.02}FA_{0.85})PbI_3$	26 µL CsPbI3 + 4 µL MAPbI3 + 170 µL FAPbI3
No.28	$(MA_{0.1}FA_{0.9})PbI_3$	20 µL MAPbI3 + 180 µL FAPbI3	No.60	$(Rb_{0.03}Cs_{0.13}MA_{0.02}FA_{0.85})PbI_3$	6 µL RbPbI3 + 20 µL CsPbI3 + 4 µL MAPbI3 + 170 µL FAPbI3

No.29	$(MA_{0.15}FA_{0.85})PbI_3$	30 µL MAPbI3 + 170 µL FAPbI3	No.61	$(Cs_{0.05}MA_{0.15}FA_{0.8})PbI_3$	10 µL CsPbI3 + 30 µL MAPbI3 + 160 µL FAPbI3
No.30	$(MA_{0.2}FA_{0.8})PbI_3$	40 µL MAPbI3 + 160 µL FAPbI3	No.62	$(Cs_{0.1}MA_{0.1}FA_{0.8})PbI_3$	20 µL CsPbI3 + 20 µL MAPbI3 + 160 µL FAPbI3
No.31	$(MA_{0.3}FA_{0.7})PbI_3$	60 µL MAPbI3 + 140 µL FAPbI3	No.63	$(Cs_{0.15}MA_{0.05}FA_{0.8})PbI_3$	30 µL CsPbI3 + 10 µL MAPbI3 + 160 µL FAPbI3
No.32	$MAPbI_3$	200 µL MAPbI3	No.64	$(Rb_{0.05}Cs_{0.05}MA_{0.1}FA_{0.8})PbI_3$	10 µL RbPbI3 + 10 µL CsPbI3 + 20 µL MAPbI3 + 160 µL FAPbI3

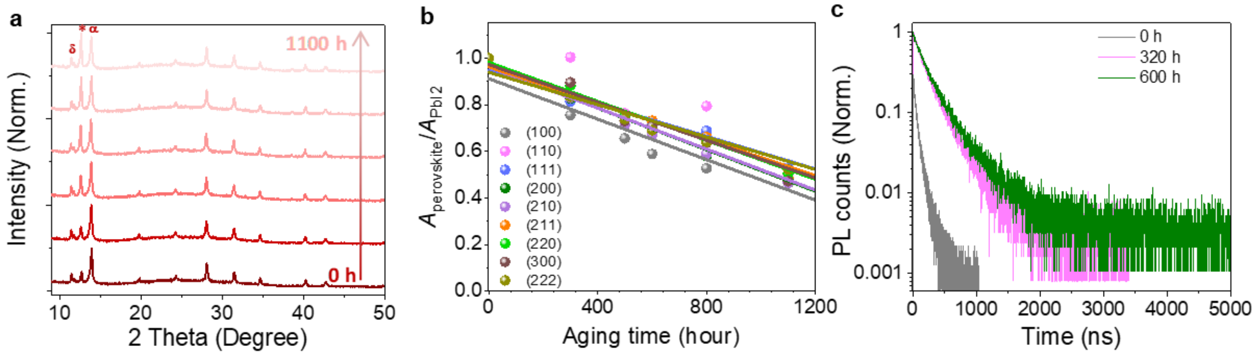

Supplementary Fig. 3. a The evolution of the XRD patterns at increasing ageing time for $FAPbI_3$ at 85℃. Symbol **δ/*/α** indicates δ-$FAPbI_3$, decomposition product PbI_2, α-$FAPbI_3$ respectively. The decomposition process is manifested by more and more pronounced PbI_2 peak. **b** The ratio of α-$FAPbI_3$ perovskite to PbI_2 as a function of ageing time at 85℃. The areas under the (001) peaks at 2θ=13.9° and 12.6° are used to represent the amount of α-$FAPbI_3$ perovskite perovskite and PbI_2, respectively. A similar calculation was also applied to other facets of perovskite. Compared with the degradation rate obtained from absorbance, the faster degradation derived from the XRD peak area is mainly because we adopted the ratio value of perovskite to PbI_2, not the absolute value of perovskite, to exclude the intensity variation in *ex situ* measurements. **c** Time-resolved photoluminescence of $FAPbI_3$ after thermal ageing for over 900 hours at 85 ℃.

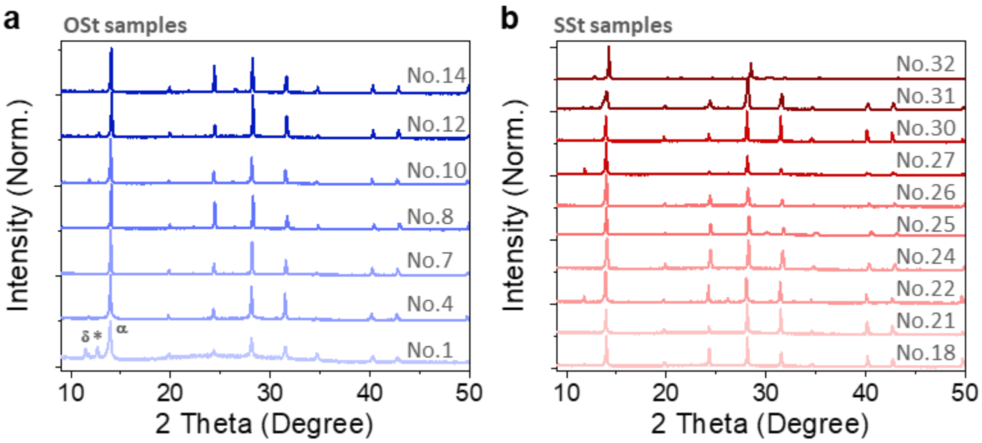

Supplementary Fig. 4. a-b XRD patterns of some typical drop-cast samples. XRD patterns of perovskites with over-stoichiometric doping referred as OSt (*e.g.*, excess MAI/CsI) **(a)** and standard stoichiometric doping referred as SSt (*e.g.*, No. 30: $MA_{0.2}FA_{0.8}PbI_3$) **(b)**. Symbol **δ/*/α** indicates δ-$FAPbI_3$, decomposition product PbI_2, α-$FAPbI_3$ respectively.

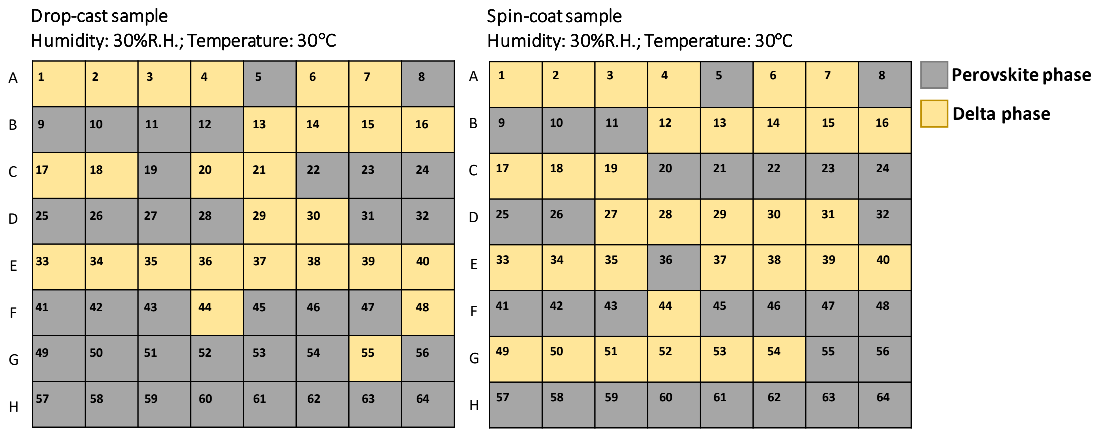

Supplementary Fig. 5. The phase map of the 64 perovskites after storing the samples in a climate chamber with humidity of 30% RH for 5 minutes. The phase map shows that the abilities of Cs doping to stabilize the α phase is stronger than MA. Note that all the samples can maintain the α phase when the humidity is below 25% RH.

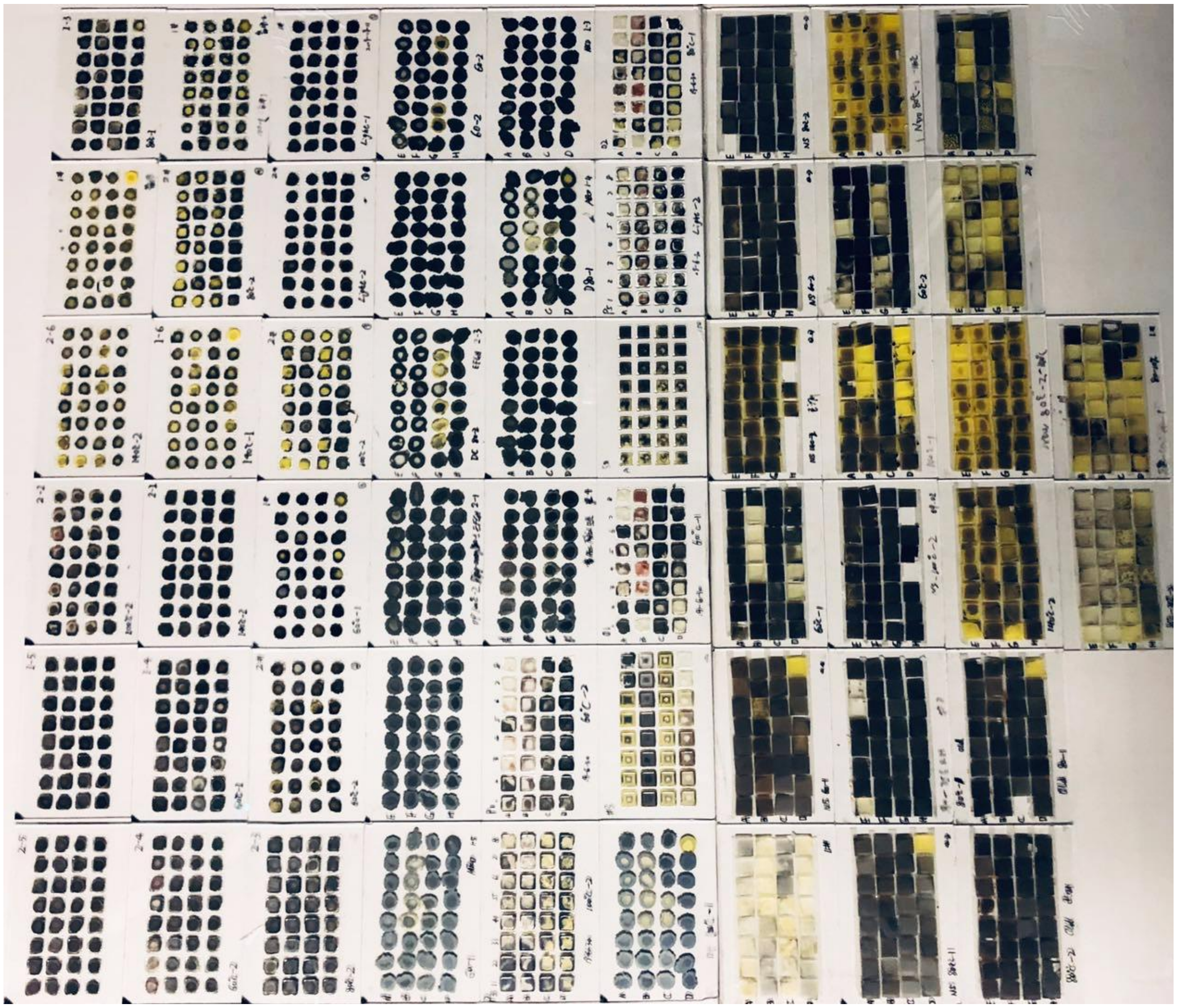

Supplementary Fig. 6. Photograph of the samples fabricated *via* the high-throughput robot. The picture was taken after performing the degradation test. Some pixels show a white colour due to the α–δ phase transition.

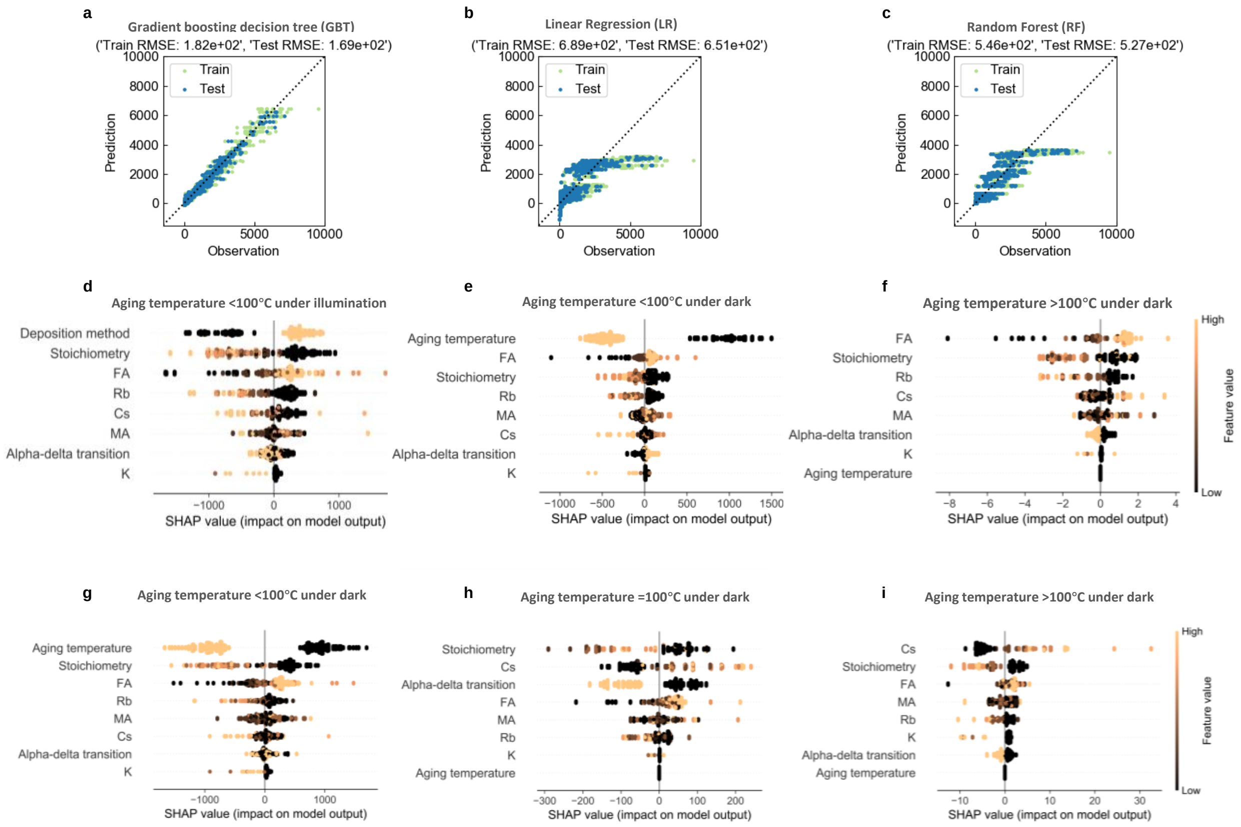

Supplementary Fig. 7. a-c The comparison between prediction and observation using GBT regression **(a)**, LR regression **(b)** and RF regression **(c)** with 80%:20% train:test set. **d** The feature importance ranking based on GBT and SHAP analysis for the T_{80} lifetime of all the samples including spin-coating and drop-casting under illumination. **e-f** The feature importance ranking based on GBT and SHAP analysis for spin-coating samples (85°C and 140 °C). **g-i** The feature importance ranking based on GBT and SHAP analysis for drop-casting samples (85°C, 100 °C and 140 °C). In the figure legend: Stoichiometry is set as 1 for over-stoichiometric condition (OSt), Deposition_method is set as 1 for drop-casting method.

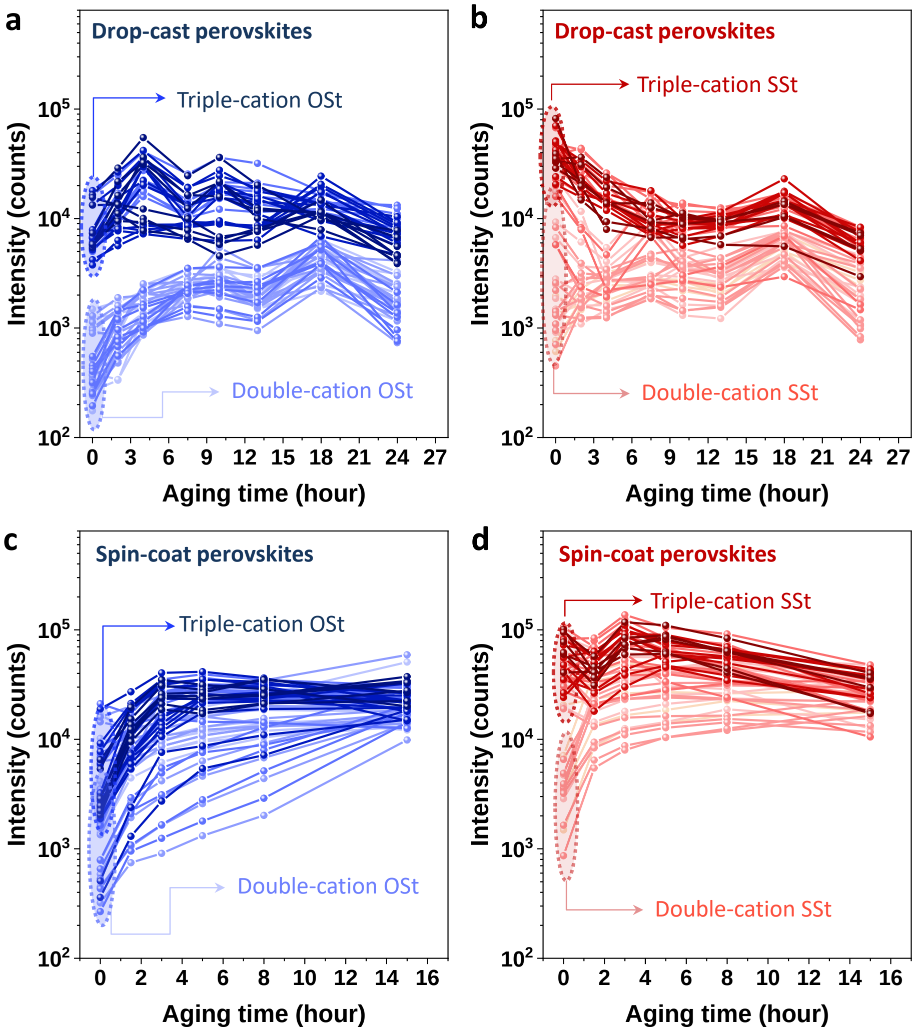

Supplementary Fig. 8. a-b PL intensity change with ageing time under 140°C for drop-cast over-stoichiometric perovskites with excess halide salts referred as OSt **(a)** and standard-stoichiometric perovskites referred as SSt **(b)**; **c-d** for spin-coated OSt perovskites with excess halide salts **(c)** and SSt perovskites **(d)**. Statistically, the PL intensity of the SSt perovskites is almost one order of magnitude larger than that of SSt samples for both double-cation and triple-cation perovskites. Most perovskites show an initial increase in PL intensity, except for triple-cation perovskites *via the* drop-cast method.

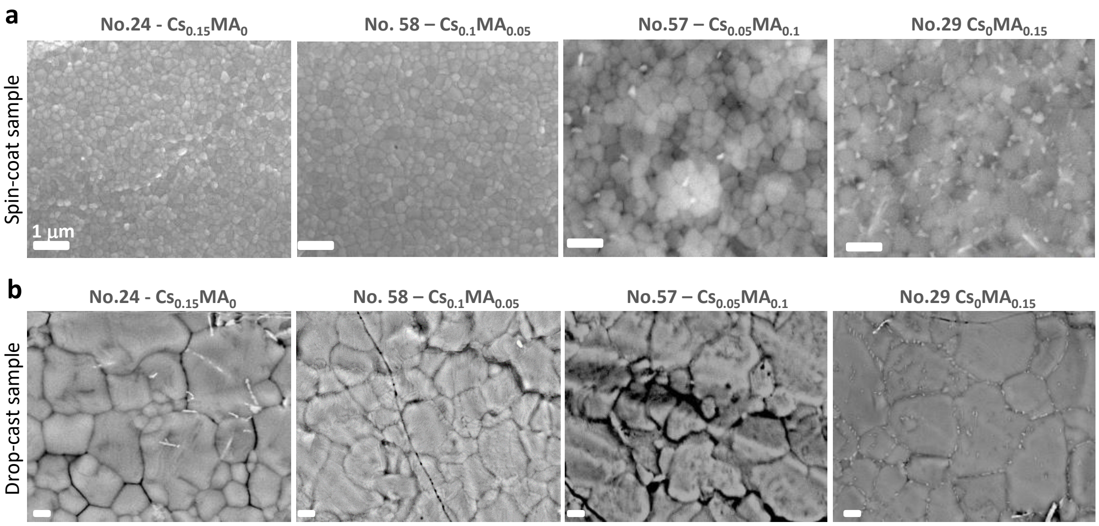

Supplementary Fig. 9. Scanning electron microscopy images of **(a)** spin-coated samples and **(b)** drop-cast samples. Scale bar: 1 μm.

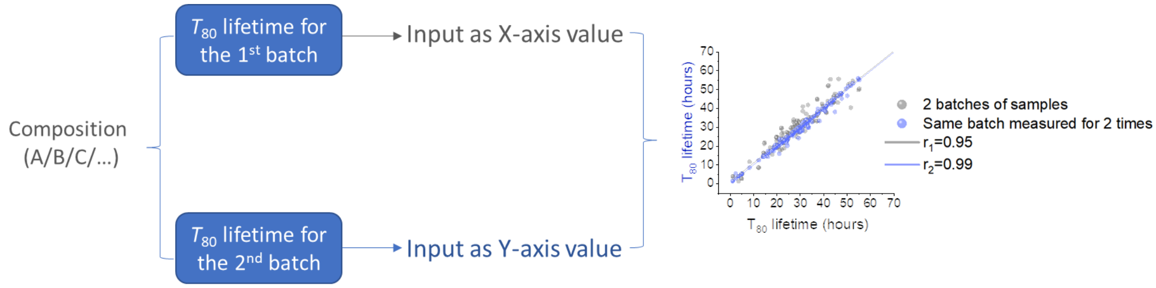

Supplementary Fig. 10. Schematic of the self-correlation to validate the reproducibility of the high-throughput system. The self-correlation is defined by correlating T_{80} lifetime of 2 batches of samples with identical compositions, processing and ageing condition.

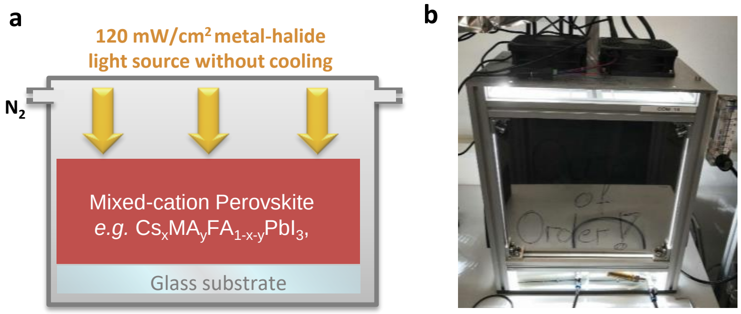

Supplementary Fig. 11. a Schematic of the photostability test. **b** Photograph of the homemade setup equipped with 5 metal halide lamps inside the chamber. The sample box is encapsulated with a glass cover. Fresh N_2 continuously flows through the sample box.

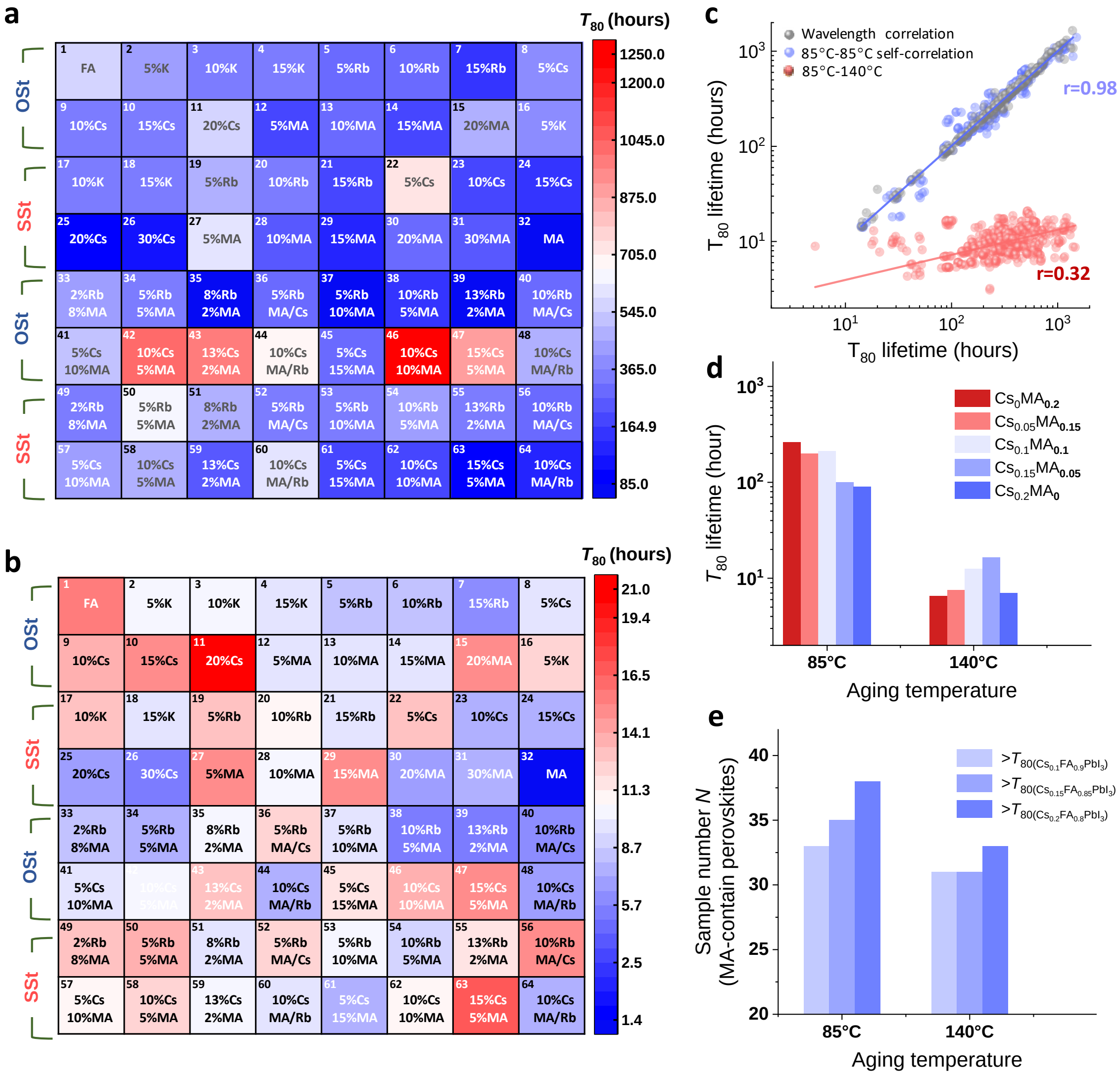

Supplementary Fig. 12. a-b The colour map of the T_{80} lifetime for the 64 spin-coat perovskites with antisovlent quenching, aged in climate chambers preset at 85℃ with 10% RH in the dark **(a)** and 140℃ with 10% RH in the dark **(b). c** The correlation plot of T_{80} at 85 ℃ against T_{80} at 140 ℃. Linear fitting was used to fit all the statistical data to obtain the Pearson correlation coefficients. **d** T_{80} lifetime *versus* ageing temperature for a series of $Cs_xMA_{0.2\text{-}x}FA_{0.8}PbI_3$ perovskites (standard-stoichiometric). **e** The sample counts of MA-containing perovskites that have longer lifetimes than 3 typical MA-free perovskites as a function of ageing temperature.

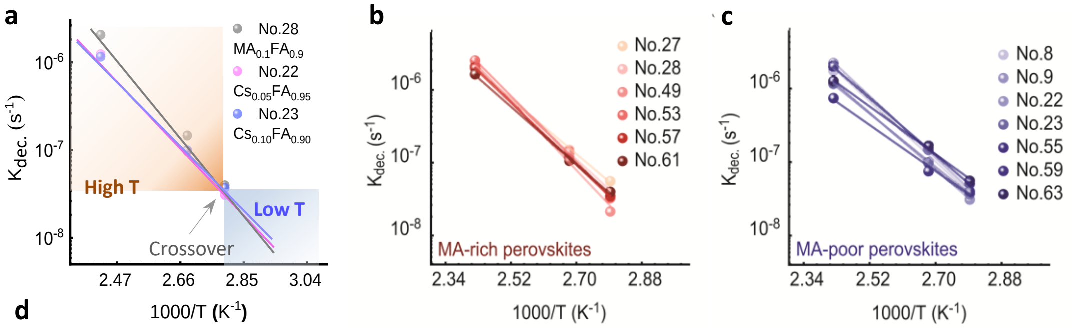

d

Table : Summary of E_a and rate constant K_0 in decomposition kinetics of perovskite. In Arrhenius equation: $K=K_o \exp(-E_a/K_BT)$, where K is the decomposition rate, K_o is the rate constant, E_a is the activation energy.

Serial Number	Composition	E_a (eV)	lg(K_0) (s^{-1})
No.1	$FAPbI_3$	0.92	5.3
No.32	$MAPbI_3$	1.16	9.4
No.27	$MA_{0.05}FA_{0.95}PbI_3$	0.84	4.5
No.28	$MA_{0.1}FA_{0.9}PbI_3$	0.97	5.4
No.49	$MA_{0.08}Rb_{0.02}FA_{0.9}PbI_3$	1.06	7.1
No.53	$MA_{0.1}Rb_{0.05}FA_{0.85}PbI_3$	0.99	6.6
No.57	$MA_{0.1}Cs_{0.05}FA_{0.85}PbI_3$	0.96	5.8
No.61	$MA_{0.15}Cs_{0.05}FA_{0.8}PbI_3$	0.88	4.8
No.8	$FAPbI_3$ (excess 5%CsI)	0.90	5.3
No.9	$FAPbI_3$ (excess 10%CsI)	0.92	5.6
No.22	$Cs_{0.05}FA_{0.95}PbI_3$	0.86	4.5
No.23	$Cs_{0.1}FA_{0.9}PbI_3$	0.79	3.8
No.55	$Rb_{0.13}MA_{0.02}FA_{0.85}PbI_3$	0.85	4.7
No.59	$Cs_{0.13}MA_{0.02}FA_{0.85}PbI_3$	0.71	2.3
No.63	$Cs_{0.15}MA_{0.05}FA_{0.8}PbI_3$	0.72	2.9

Supplementary Fig. 13. a k_{dec}-1000/T plot of No. 28 and No. 22/23 (MA-free perovskites) in logarithmic coordinates, where k_{dec} is the decomposition rate (s^{-1}) and 1000/T is the reciprocal of the ageing temperature (K^{-1}). The data are fitted using equation (2). **b-c** k_{dec}-1000/T plot of MA-rich perovskites (MA concentration >5%) and MA-poor perovskites (MA concentration <5%). **d** A summary of the activation energies and pre-exponential factors for some typical MA-rich and Cs/Rb-rich perovskites, obtained by exponentially fitting the data in Fig. 2.

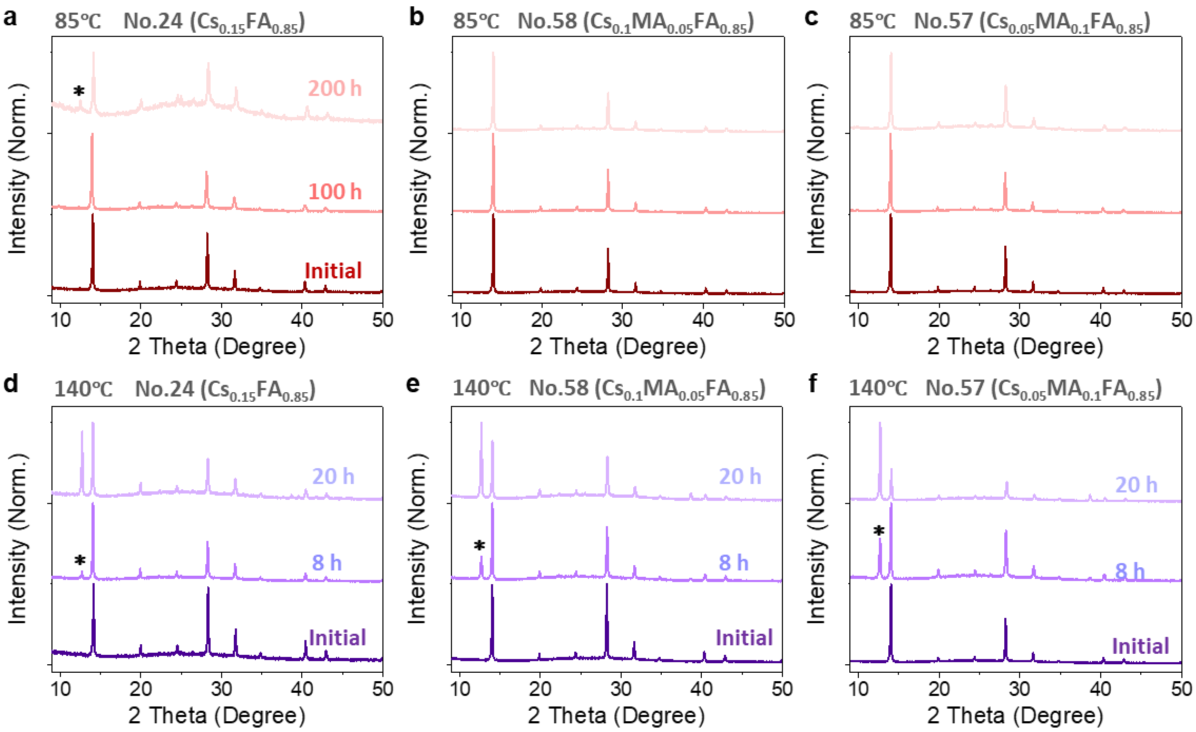

Supplementary Fig. 14. a-c XRD patterns of 3 typical perovskites with increasing ageing time at 85°C. **d-f** XRD patterns of 3 typical perovskites with increasing ageing time at 140°C. Symbol '*' indicates PbI_2 in the sample.

a

Summary of the target composition for the selected 32 spin-coat samples for photo-stability evaluation.
Blue and red color indicate doping with overstoichiometry and standard-stoichiometry, respectively

1	2	3	4	5	6	7	8
$FAPbI_3$	MAI-5%	MAI-10%	MAI-15%	KI-15%	RbI-5%	RbI-10%	RbI-15%
9	10	11	12	13	14	15	16
CsI-5%	CsI-10%	CsI-15%	MAI/RbI-5%/10%	MAI/RbI-10%/5%	MAI/CsI-5%/15%	MAI/CsI-10%/10%	MAI/CsI-15%/5%
17	18	19	20	21	22	23	24
$MA_{0.05}FA_{0.95}PbI_3$	$MA_{0.1}FA_{0.9}PbI_3$	$MA_{0.15}FA_{0.85}PbI_3$	$K_{0.15}FA_{0.85}PbI_3$	$Rb_{0.05}FA_{0.95}PbI_3$	$Rb_{0.1}FA_{0.9}PbI_3$	$Rb_{0.15}FA_{0.85}PbI_3$	$Cs_{0.05}FA_{0.95}PbI_3$
25	26	27	28	29	30	31	32
$Cs_{0.1}FA_{0.9}PbI_3$	$Cs_{0.15}FA_{0.85}PbI_3$	$MA_{0.05}Rb_{0.1}FA_{0.85}PbI_3$	$MA_{0.1}Rb_{0.05}FA_{0.85}PbI_3$	$MA_{0.05}Cs_{0.15}FA_{0.8}PbI_3$	$MA_{0.1}Cs_{0.1}FA_{0.8}PbI_3$	$MA_{0.15}Cs_{0.05}FA_{0.8}PbI_3$	$MAPbI_3$

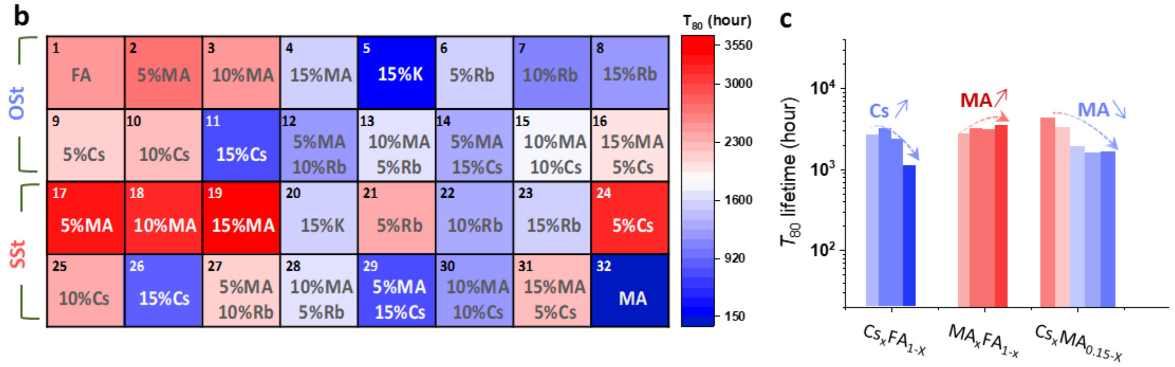

Supplementary Fig. 15. a Summary of the compositions of spin-coated samples selected for the photostability test. **b** The colormap of the T_{80} lifetime for the 32 perovskites under light illumination (metal halide lamp) of 120 mW cm^{-2} intensity at 60°C. **c** T_{80} lifetime of a series of $Cs_xMA_yFA_{1-x-y}PbI_3$ perovskites under light illumination. For $Cs_xFA_{1-x}PbI_3$ and $MA_xFA_{1-x}PbI_3$, the x values equal 5%, 10%, 15%, 20% and 30% from left to right; for $Cs_xMA_{0.15-x}$, the x values equal 0%, 5%, 10%, 13% and 15% from left to right.

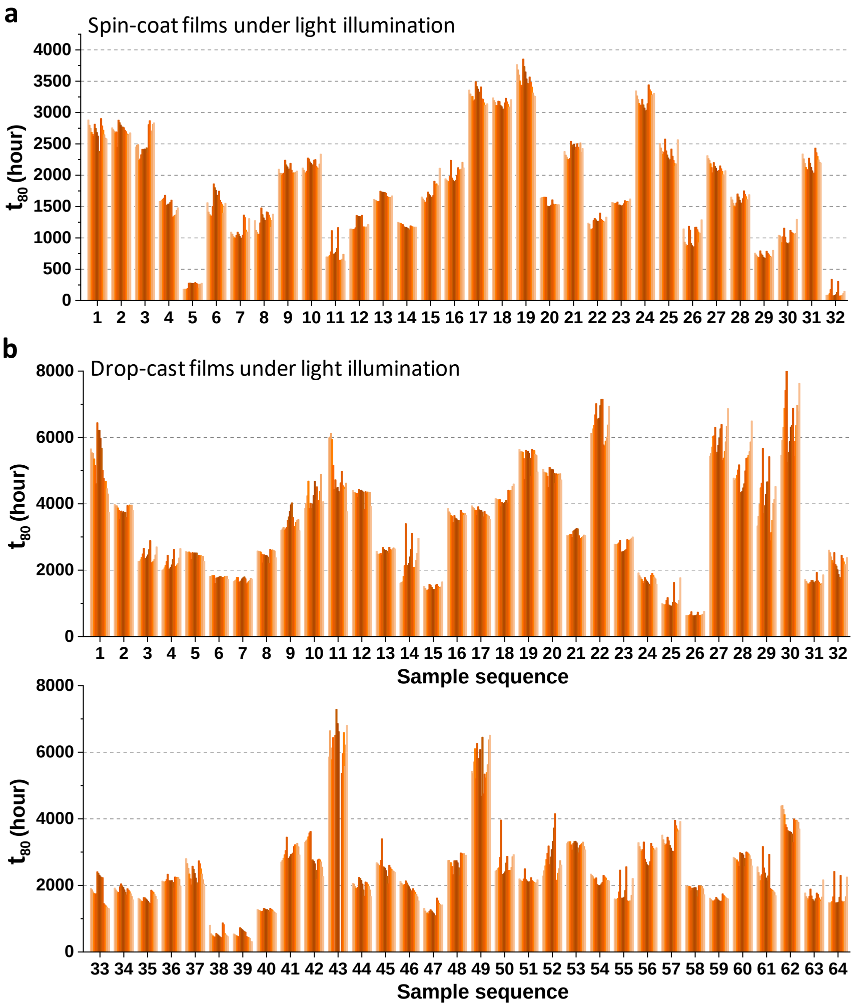

Supplementary Fig. 16. a The histogram of the T_{80} lifetimes for the 32 spin-coated perovskites. **b** The histogram of T_{80} lifetimes of the 64 drop-cast perovskites under light illumination (metal halide lamp) of 120 mW cm^{-2} intensity at 60°C.

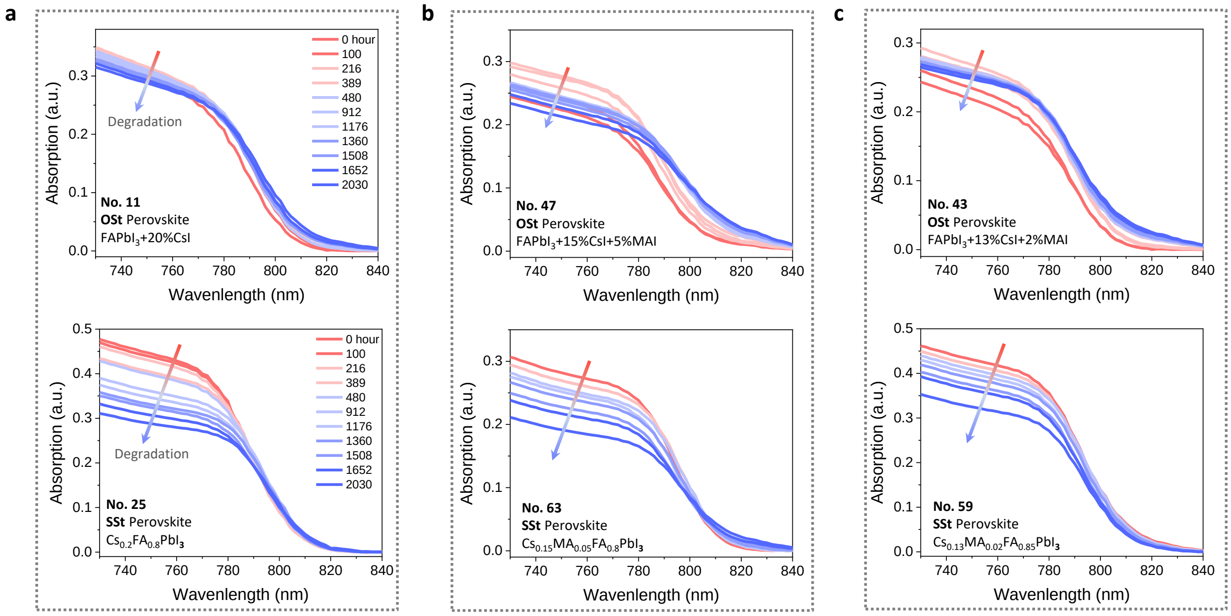

Supplementary Fig. 17. Comparison of the absorption spectra of over-stoichiometric (OSt) perovskites and standard-stoichiometric (SSt) perovskites during degradation under light illumination. **a** Evolution of the absorption spectra of No. 11 (OSt sample) and No. 25 (SSt sample); **b** for No. 47 (OSt sample) and No. 63 (SSt sample); and **c** for No. 43 (OSt sample) and No. 59 (SSt sample). OSt samples generally present a redshift during degradation, yet this shift is negligible in SSt samples due to the absence of excess halide salts.

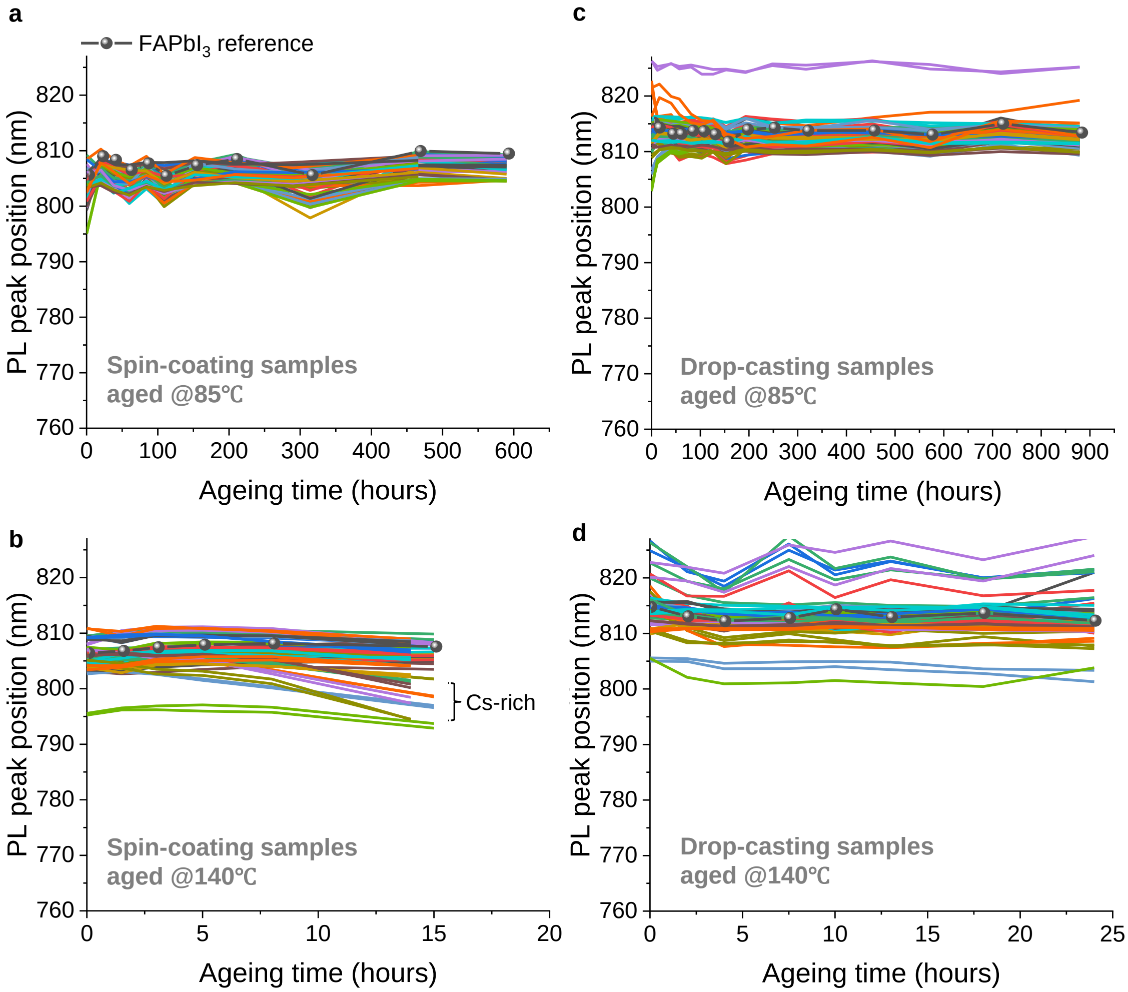

Supplementary Fig. 18. Comparison of the photoluminscence spectra of standard-stoichiometric (SSt) perovskites during degradation. a-b Evolution of the PL peak position for spin-coating SSt perovskites, inlcuding double-cation and triple-cation perovskites during decomposition process at 85 and 140°C; **c-d** Evolution of the PL peak position for drop-casting SSt perovskites, inlcuding double-cation and triple-cation perovskites during decomposition process at 85 and 140°C.

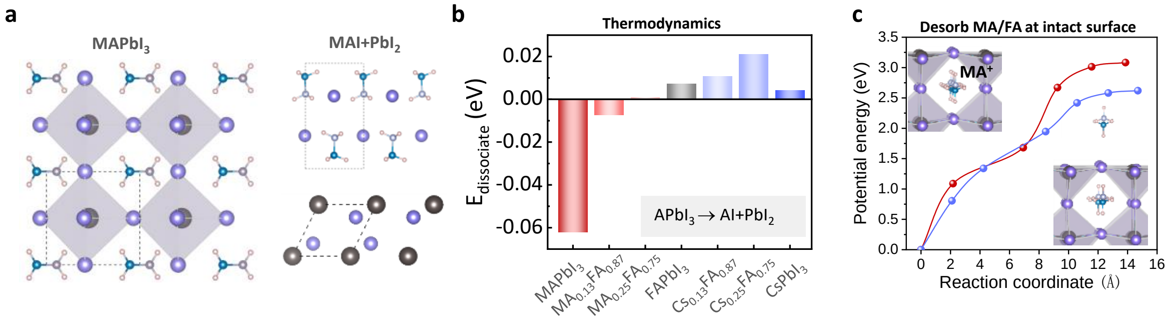

Supplementary Figure 19. a The crystal structures of $MAPbI_3$ and its decomposition products, PbI_2 and MAI. **b** Bar chart of the dissociation energies of $FAPbI_3$, $MAPbI_3$, $CsPbI_3$ and $FAPbI_3$-based mixed-cation perovskites. **c** The potential energy curve *vs.* reaction coordinate for the desorption of MA/FA on the intact surface of $MAPbI_3$/$FAPbI_3$.

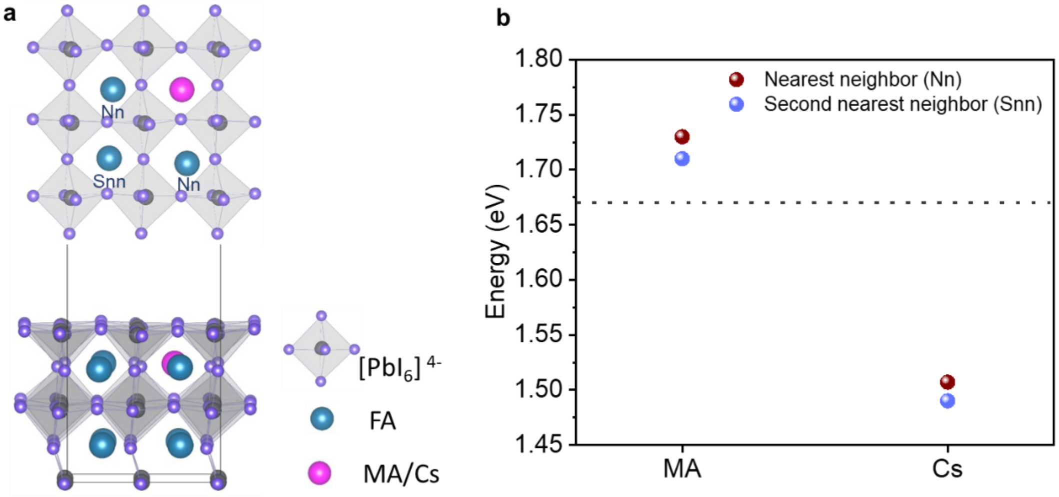

Supplementary Figure 20. a The crystal tructure of $A_{0.13}FA_{0.87}PbI_3$ alloy (A= MA,Cs). The FA molecules on the surface can be divided into the nearest neighbor (Nn) and the second nearest neighbor (Snn). **b** Desorption energy of FA molecules at different positions in $A_{0.13}FA_{0.87}PbI_3$ alloy. The dotted line represents the desorption energy of FA in pristine $FAPbI_3$ without doping.

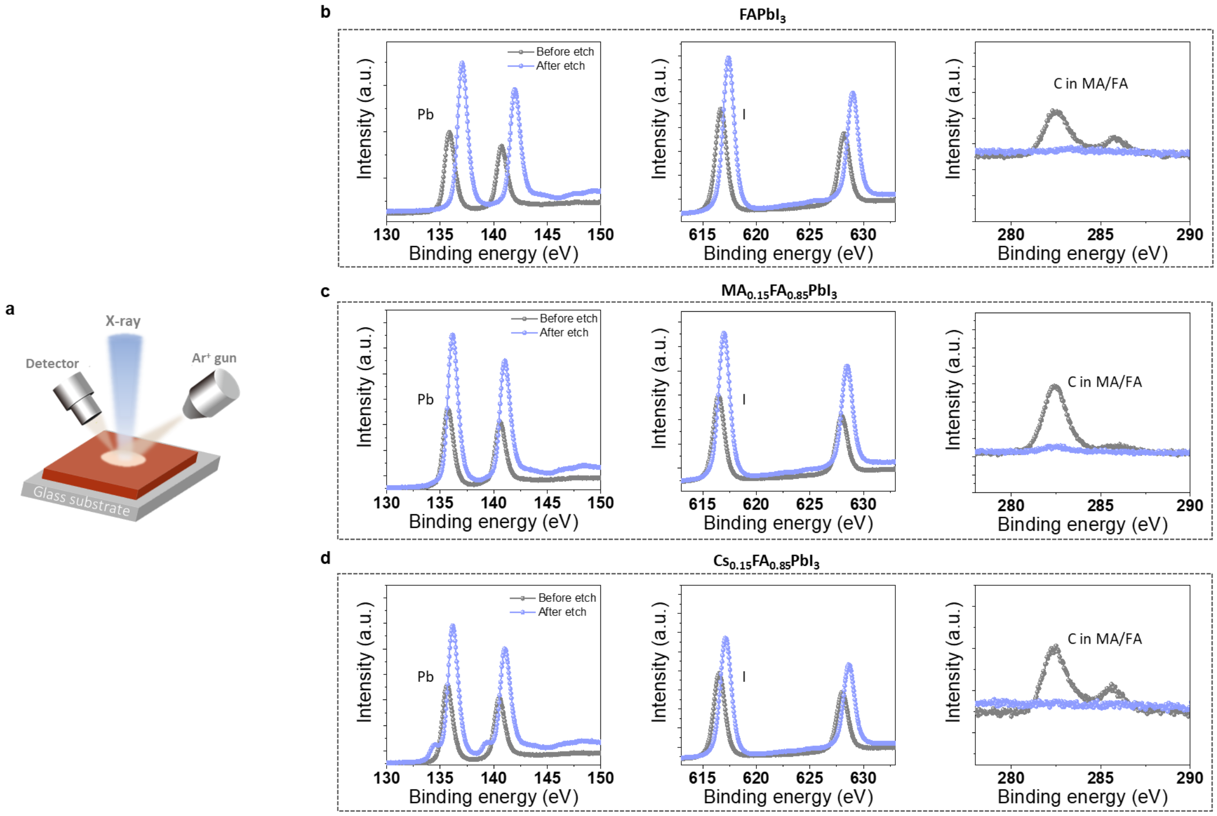

Supplementary Figure 21. a The schematic diagram of the setup of X-ray photoelectron spectroscopy (XPS) with ion-beam etching. **b** The XPS patterns of $FAPbI_3$ before and after 10 seconds Ar^+ etching with 2 KV acceleration voltage and 20 uA electron neutralizer. **c** The XPS patterns of $MA_{0.15}FA_{0.85}PbI_3$ before and after 10 seconds Ar^+ etching with above condition. **d** The XPS patterns of $Cs_{0.15}FA_{0.85}PbI_3$ before and after 10 seconds Ar^+ etching with above condition.

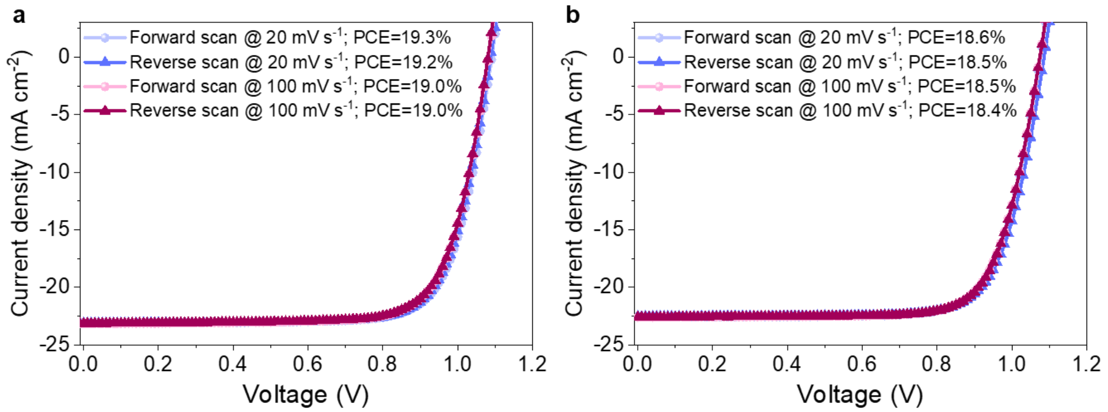

Supplementary Figure 22. Current density-voltage (*J*-*V*) curves for $Cs_{0.1}MA_{0.05}FA_{0.85}PbI_3$ **(a)** and $Cs_{0.05}MA_{0.1}FA_{0.85}PbI_3$ **(b)** perovskite solar cells with forward scan from -0.1 V to 1.2 V and reverse scan from 1.2 V to -0.1 V at different scanning rate.

Supplementary Table 3. Report of performance parameter of five typical devices with different compositions

**Composition**	**V_{oc} (V)**	**J_{sc} (mA cm^{-2})**	**FF (%)**	**PCE (%)**
$FAPbI_3$	1.09	21.7	70	16.6
$Cs_{0.15}MA_0FA_{0.85}PbI_3$	1.06	22.6	74	17.7
$Cs_{0.10}MA_{0.05}FA_{0.85}PbI_3$	1.08	22.9	78	19.3
$Cs_{0.05}MA_{0.10}FA_{0.85}PbI_3$	1.07	23.3	76	18.8
$Cs_0MA_{0.15}FA_{0.85}PbI_3$	1.05	21.9	76	17.4

Supplementary Table 3. A summary of the parameter of photovoltaic performance.

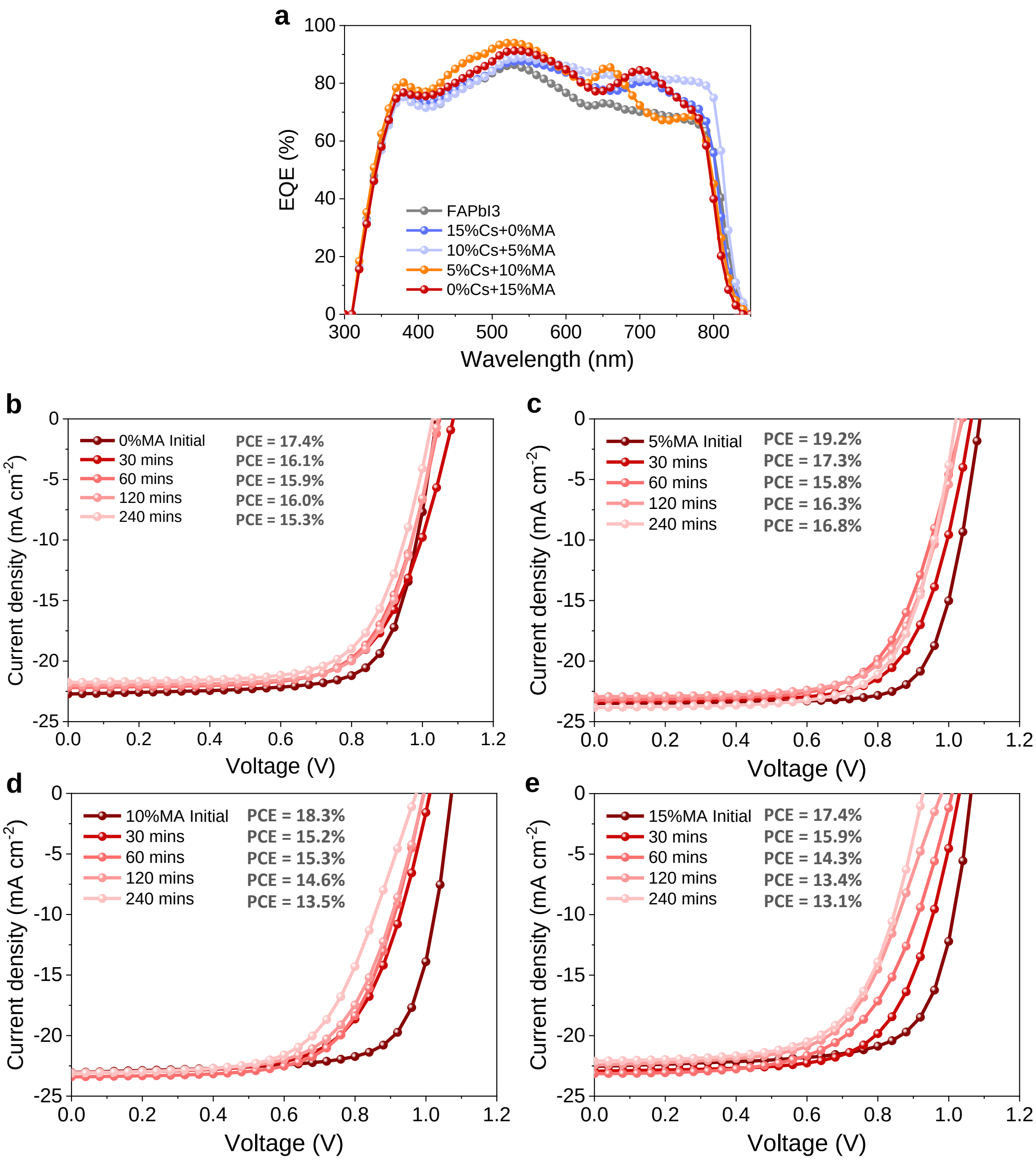

Supplementary Figure 23. a External quantum efficiency of $Cs_xMA_{0.15\text{-}x}FA_{0.85}PbI_3$ perovskite solar cells with integrated current density of 20.7/21.8/22.8/21.9/21.8 mA cm^{-2} for $FAPbI_3$/0%MA/5%MA/10%MA/15%MA respectively. **b-e** Current density-voltage (J-V) curves for $Cs_xMA_{0.15\text{-}x}FA_{0.85}PbI_3$ perovskite solar cells aged at 140°C for different times, measured with a scanning rate of 20 mV s^{-1} from −0.1 to 1.2 V. (A) x=0, 0.05, 0.1, and 0.15 are denoted as 0% MA **(b)**, 5% MA **(c)**, 10% MA **(d)**, and 15% MA **(e),** respectively. The devices are aged at 140°C before deposition of the hole transporting layer.

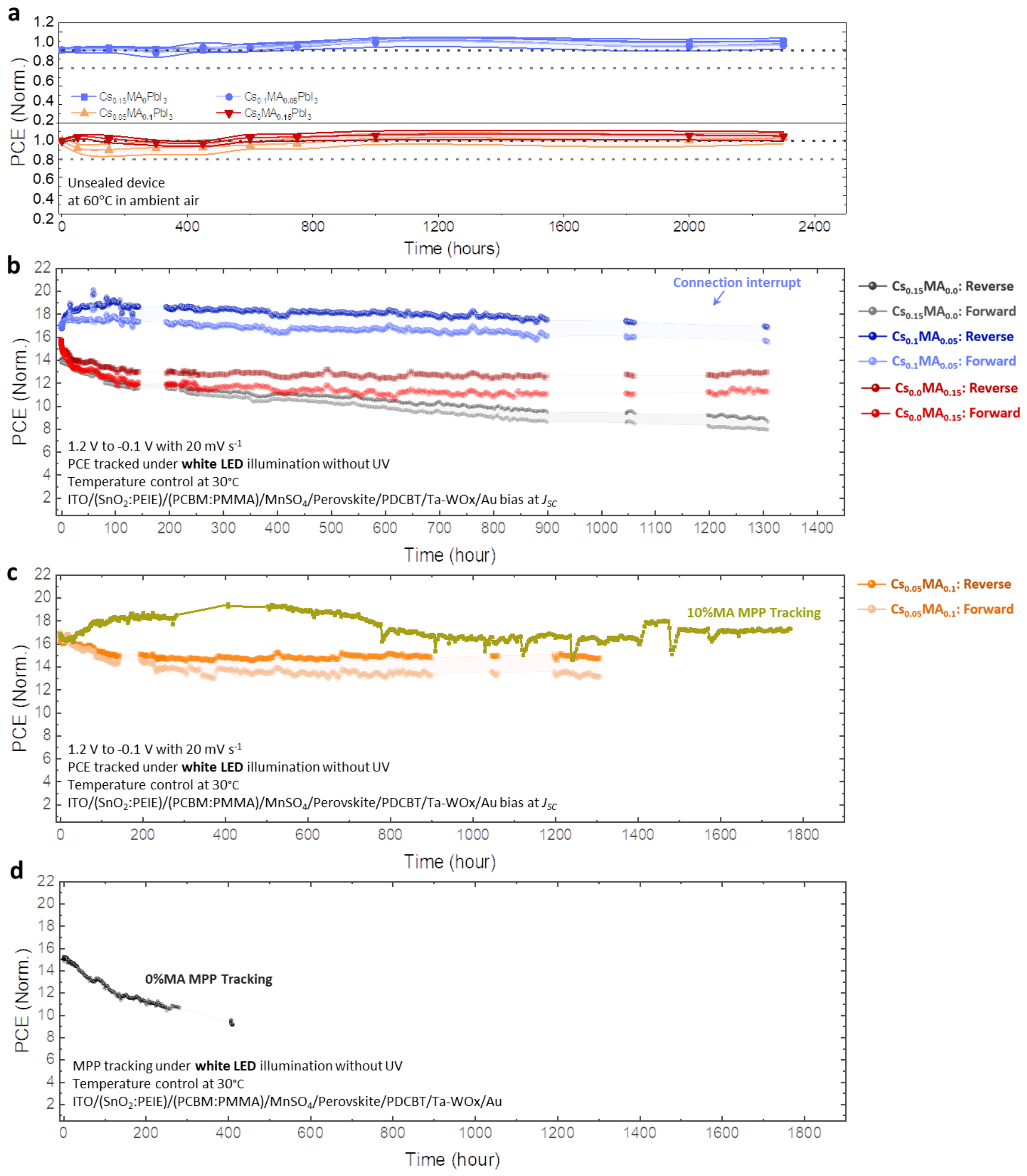

Supplementary Figure 24. a Statistical PCE change during the ageing process at 60°C in ambient air. The humidity was approximately 35% RH. **b** Long-term stability of $Cs_xMA_{0.15\text{-}x}FA_{0.85}PbI_3$ (x=0, 5%, and 15%) perovskite solar cells tested under 100 mW cm^{-2} white LED illumination. For each sample, the efficiency was recorded at a scanning rate of 20 mV s^{-1} from 1.2 to −0.1 V as the reverse scan and from -0.1 V to 1.2 V as the forward scan. **c** Long-term stability for x=10% with MPP tracking. **d** Long-term stability for x=0% with MPP tracking. The efficiency value is based on the stabilized efficiency biased at the maximum power point.

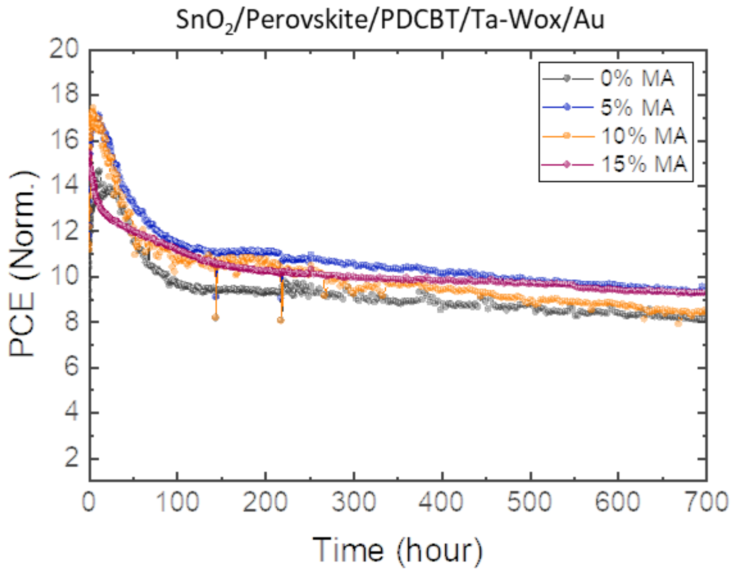

Supplementary Figure 25. Long-term stability of $Cs_xMA_{0.15\text{-}x}FA_{0.85}PbI_3$ (x=0%, 5%, 10% and 15%) perovskite solar cells with device structure of SnO_2/Perovskite/PDCBT/Ta-WO_x/Au, under 100 mW cm^{-2} white LED illumination. The efficiency was recorded at a scanning rate of 20 mV s^{-1} from 1.2 to −0.1 V.

Supplementary Table 4. A summary of previous studies on the device stability of mixed-cation perovskite solar cells.

Supplementary Table 4: Summary of device stability on perovskites (MA^+: $CH_3NH_3^+$; FA^+: $NH_2CHNH_2^+$)

Aging condition	Composition and processing		Conclusion	Reference
85°C-light in N_2	$Rb_{0.05}(Cs_{0.05}MAFA)_{0.95}Pb(I_{0.83}Br_{0.17})_3$-based devices *via* 1-step spin-coating	MA-containing	5% loss after 500 hours	Science 354, 206-209 (2016).
30°C-light in N_2	$Cs_x(MA_{0.17}FA_{0.83})_{(100-x)}Pb(I_{0.83}Br_{0.17})_3$ (x=0/10)-based devices *via* 1-step spin-coating	MA-containing	10% loss after 250 hours	Energy Environ. Sci. 9, 1989-199 (2016)
30°C-light with N_2 flow	$Cs_x(MA_{0.17}FA_{0.83})_{(100-x)}Pb(I_{0.97}Br_{0.03})_3$ (x=0/1/2/3) devices *via* 2-step spin-coating	MA-containing	40% loss after 400 hours	Nat. Commun. 9, 1607 (2018)
25°C-light in air (humidity: 40%RH)	$MA_{0.15}FA_{0.85}Pb(I_{0.85}Br_{0.15})_3$-based deviceswith excess 5 mol.% RbI *via* 1-step spin-coating	MA-containing	0% loss after100 hours	Nano Energy 30, 330–340 (2016)
30°C-light in air (humidity not indicated)	$(5\text{-}AVA)_x(MA)_{1\text{-}x}PbI_3$ –based devices *via* doctor-blading	MA-containing	0% loss after 1000 hours	Science 345, 295-298 (2014)
30°C-light in N_2	$(FAPbI_3)_{0.95}(MAPbBr_3)_{0.05}$-based devices *via* 1-step spin-coating	MA-containing	5% loss after 1400 hours	Nature 567, 511–515 (2019)
25°C in air under dark	$FA_{0.15}MA_{0.85}PbI_3$-based devices *via* 2-step spin-coating and thermal evaporation	MA-containing	3% loss after 800 hours	Nat. Energy, 4: 150 (2019)
85°C-dark in N_2	$MAPbI_3$-based devices *via* 1-step spin-coating	MA-containing	5% loss after 500 hours	Nat. Commun. 10: 1161 (2019)
30°C-light in N_2	$MAPbI_3$-based devices *via* 1-step spin-coating	MA-containing	3% loss after 500 hours	Nat. Commun. 10: 1161 (2019)
65°C-light in N_2	$Cs_{0.05}MA_{0.14}FA_{0.81}PbI_{2.55}Br_{0.45}$-based devices *via* 1-step spin-coating	MA-containing	3% loss after 1200 hours	Science 365, 473–478 (2019)
85°C-dark in air	$FA_{0.83}Cs_{0.17}Pb(I_{0.83}Br_{0.17})_3$-based devices *via* 1-step spin-coating	MA-free	25% efficiency loss after 3 hours	Adv. Fun. Mater. 29, 1900466 (2019)
30°C-light in N_2	$Rb_5Cs_{10}FAPbI_3$-based devices *via* 1-step spin-coating	MA-free	22% loss after 1000h and 6% loss with surface passivation	Science 362, 449–453 (2018)
35°C-light in N_2	$Cs_{0.17}FA_{0.83}Pb(Br_{0.17}I_{0.83})_3$-based devices *via* 1-step spin-coating	MA-free	15% efficiency loss after 600 hours	Nat. Energy 5, 35-49 (2020)
25°C-light in N_2	$Cs_{0.925}K_{0.075}PbI_2Br$	MA-free	20% efficiency loss after 120 hours	Nano Lett. 17, 2028-2033 (2017)
60°C-light in N_2	$Cs_{0.1}FA_{0.9}PbI_3$	MA-free	30% efficiency loss after 220 hours	Adv. Energy Mater. 5, 1501310 (2015)
60°C-light in air	$Cs_{0.17}FA_{0.83}Pb(Br_{0.4}I_{0.6})_3$	MA-free	20% efficiency loss after 1000 hours	Nat. Energy 2, 17135 (2017)
