# Supplementary material for: Discovery of temperature-induced stability reversal in perovskites using high-throughput robotic learning
Source: Nat Commun. 2021 Apr 13;12:2191. doi: 10.1038/s41467-021-22472-x (PMC8044090; doi:10.1038/s41467-021-22472-x)
Supplement: Supplementary file 2 — Description of Additional Supplementary Files [file 41467_2021_22472_MOESM2_ESM.pdf]

Description of Additional Supplementary Files

File Name: Supplementary Data 1
Description: T_{80} lifetime for spin-coat samples

File Name: Supplementary Data 2
Description: T_{80} lifetime for drop-cast samples

File Name: Supplementary Movie 1
Description: The platform and data analysis for high-throughput robot system

File Name: Supplementary Software 1
Description: Analysis codes to extract the T_{80} lifetime and PL features
