# Supplementary material for: Discovery of temperature-induced stability reversal in perovskites using high-throughput robotic learning
Source: Nat Commun. 2021 Apr 13;12:2191. doi: 10.1038/s41467-021-22472-x (PMC8044090; doi:10.1038/s41467-021-22472-x)
Supplement: Supplementary file 7 — Solar Cells Reporting Summary [file 41467_2021_22472_MOESM7_ESM.pdf]

nature research

Corresponding author(s): Yicheng Zhao

Solar Cells Reporting Summary

Nature Research wishes to improve the reproducibility of the work that we publish. This form is intended for publication with all accepted papers reporting the characterization of photovoltaic devices and provides structure for consistency and transparency in reporting. Some list items might not apply to an individual manuscript, but all fields must be completed for clarity.

For further information on Nature Research policies, including our data availability policy, see Authors & Referees.

Experimental design

Please check: are the following details reported in the manuscript?

1. Dimensions

Item	Yes	No	Details
Area of the tested solar cells	☒	☐	Method
Method used to determine the device area	☒	☐	Method: The area of aperture mask is 0.113 cm2

2. Current-voltage characterization

Item	Yes	No	Details
Current density-voltage (J-V) plots in both forward and backward direction	☒	☐	Method
Voltage scan conditions<br>*For instance: scan direction, speed, dwell times*	☒	☐	Method:The J-V characteristics were measured from -0.1 to 1.2 V (forward scan) and 1.2 to -0.1 V (reverse scan) at a scan rate of 20 mV/s.
Test environment<br>*For instance: characterization temperature, in air or in glove box*	☒	☐	Method:The tests were tested in air at room temperature (around 25 °C)
Protocol for preconditioning of the device before its characterization	☐	☒	*Explain why this information is not reported/not relevant.*
Stability of the J-V characteristic<br>*Verified with time evolution of the maximum power point or with the photocurrent at maximum power point; see ref. 7 for details.*	☒	☐	Method:For maximum power point (MPP) tracking, the MPP tracking point is based on the reverse J-V scan from 1.2 to -0.1 V every 2-3 hours. The stabilized efficiency was obtained by fixing the bias at the MPP point for 2 minutes.

3. Hysteresis or any other unusual behaviour

Item	Yes	No	Details
Description of the unusual behaviour observed during the characterization	☐	☒	*Explain why this information is not reported/not relevant.*
Related experimental data	☒	☐	Supplementary Figure 21: The hysteresis is negligible in our fresh devices

4. Efficiency

Item	Yes	No	Details
External quantum efficiency (EQE) or incident photons to current efficiency (IPCE)	☒	☐	Supplementary Figure 20
A comparison between the integrated response under the standard reference spectrum and the response measure under the simulator	☒	☐	Supplementary Figure 20
For tandem solar cells, the bias illumination and bias voltage used for each subcell	☐	☒	not applicable

5. Calibration

Item	Yes	No	Details
Light source and reference cell or sensor used for the characterization	☒	☐	Method
Confirmation that the reference cell was calibrated and certified	☒	☐	Method

Calculation of spectral mismatch between the reference cell and the devices under test

☒ Yes ☐ No — Spectral mismatch factor of 1 is used for all the J-V measurements

6. Mask/aperture

Size of the mask/aperture used during testing

☒ Yes ☐ No — Method: The area of aperture mask is 0.113 cm2

Variation of the measured short-circuit current density with the mask/aperture area

☐ Yes ☒ No — not applicable

7. Performance certification

Identity of the independent certification laboratory that confirmed the photovoltaic performance

☐ Yes ☒ No — not relevant

A copy of any certificate(s)
Provide in Supplementary Information

☐ Yes ☒ No — not relevant

8. Statistics

Number of solar cells tested

☒ Yes ☐ No — Figure 4 in the main text

Statistical analysis of the device performance

☒ Yes ☐ No — Figure 4 in the main text

9. Long-term stability analysis

Type of analysis, bias conditions and environmental conditions
For instance: illumination type, temperature, atmosphere humidity, encapsulation method, preconditioning temperature

☒ Yes ☐ No — Method
